# Characterization of trehalose-6-phosphate synthase gene family in linseed (*Linum usitatissimum* L.) and its potential implications in flowering time regulation

**DOI:** 10.1186/s12870-025-07559-7

**Published:** 2025-11-17

**Authors:** Ankit Saroha, Daniya Shahid, J. Aravind, Sneha Murmu, Vikender Kaur, S. Rajkumar, Abhishek Sengupta, Dhammaprakash Pandhari Wankhede

**Affiliations:** 1 ICAR-National Bureau of Plant Genetic Resources, New Delhi, India; 2 Amity Institute of Biotechnology, Amity University, Noida, Uttar Pradesh India; 3 ICAR-Indian Agricultural Statistics Research Institute, New Delhi, India

**Keywords:** Flaxseed, Trehalose-6-phosphate synthase, Gene family, Comparative genomics, Flowering time

## Abstract

**Background:**

Linseed is an important oilseed crop with diverse applications in the food, nutraceutical, oil, and paint industries. Flowering time is a critical trait in linseed, as it greatly influences seed yield potential and quality across various agro-ecological zones. Trehalose-6-phosphate synthase (TPS) genes have been implicated in the regulation of flowering time in plants. Therefore, a comprehensive analysis of the TPS gene family in linseed, using comparative genomics and bioinformatics approaches, is essential for elucidating the genetic mechanisms underlying flowering time regulation in this crop.

**Results:**

A total of 18 LuTPS genes, including several paralogs, were identified in the linseed genome and clustered into two distinct groups. Gene expression analysis in developing floral buds, flower, and vegetative tissues revealed that most TPS genes exhibited basal expression levels. However, *LuTPS6.1, LuTPS6.2, LuTPS10.1, LuTPS1.1, LuTPS1.2, LuTPS7.2, LuTPS7.3, LuTPS7.4*, and *LuTPS8.*2 showed significantly higher expression and strong correlation with key flowering-related genes such as *FT, FUL,* and *SOC1*. Allelic variation analysis using early- and late-flowering linseed accessions revealed trait-specific SNPs in *LuTPS6.1* and *LuTPS10.2*. A comprehensive analysis of cis-regulatory elements *(CREs*) in the promoter regions of *LuTPS* genes, compared to the entire linseed genome, identified several *CREs* that were significantly enriched in *TPS* gene promoters, as well as those that were consistently present across all *LuTPS* gene promoters. Furthermore, the genome-wide syntenic network analysis involving linseed and nine other plant genomes provided valuable insights into TPS-associated syntelogs and the evolutionary dynamics and expansion of the TPS gene family. The physical proximity of *TPS* genes to known flowering time QTLs/QTNs is also discussed.

**Conclusion:**

This study, with TPS gene family characterization, gene expression and allelic variation highlights the potential role of the *TPS* genes in the regulation of flowering time in linseed. The identified enriched *CREs *in the promoters of TPS genes would be crucial to understand the regulation of TPS genes in growth, development and stress response. TPS-associated syntelogs provided valuable insights into the evolutionary history and expansion of the TPS gene family. Collectively, these findings represent a significant step toward understanding the complex genetic regulation of flowering time in linseed.

**Supplementary Information:**

The online version contains supplementary material available at 10.1186/s12870-025-07559-7.

## Background

Linseed, also known as flaxseed, (*Linum usitatissimum* L.) (2n = 2x = 30) is an annual herbaceous plant from the Linaceae family and is notable for its dual-use potential in food and industry [1]. Linseed is cultivated globally, and countries including Canada, Kazakhstan, Russia, China, USA, and India are the major producers. The seeds are rich in α-linolenic acid (ALA), a polyunsaturated omega-3 fatty acid, as well as lignans and dietary fiber, contributing to their growing use in nutraceuticals and functional foods [2]. Industrially, linseed is a key raw material in the production of linseed oil, which is extensively used in the manufacture of paints, varnishes, inks, and linoleum flooring due to its quick-drying and polymer-forming properties [2]. There is renewed interest in linseed reflecting the global trade of $1.44B in 2023 (https://oec.world/en/profile/hs/linseed).

Linseed exhibits two major morphotypes, ‘oilseed type’, characterized by a shorter, bushier growth habit, and ‘fiber type’, which has a taller, slender morphology optimized for fiber extraction [3, 4]. Flowering time is a critical agronomic trait in linseed, with early flowering and maturity being desirable particularly for the oilseed types, under rainfed conditions and rice fallow systems, such as those prevalent in India [5]. It also avoids abiotic stress such as terminal drought and heat, which have a negative impact on seed set, yield, and oil quality [6]. Early flowering is also advantageous to avoid frost and for expanding the cultivation range of flaxseed into cooler regions, including the northern prairies of Canada [7–9].

Flowering is a complex, quantitative trait influenced by genetic, environmental, and epigenetic factors. The intricate genetic network that determines initiation of flowering depends on environmental (day length, temperature, etc.) and endogenous signals (hormones and carbohydrate status, etc.) [10]. Six major flowering pathways have been identified in *Arabidopsis*, which are vernalization, photoperiod, autonomous, thermosensory, age and gibberellin (GA) pathways [11]. In the photoperiod pathway, flowering is regulated by day length and quality of light perceived. These pathways involve the key players such as CONSTANS (CO), *FLOWERING LOCUS T* (*FT*), *SUPPRESSOR OF OVEREXPRESSION OF CONSTANS 1* (*SOC1*), *LEAFY* (*LFY*), GIGANTEA (GI), FLAVIN-BINDING, KELCH REPEAT, F-BOX 1 (FKF1), *APETALA1* (*AP1*), FLOWERING LOCUS C (FLC), etc. [11–14].

In addition to the canonical flowering pathways and genes, there is strong evidence supporting the role of carbohydrates such as sucrose, glucose, and trehalose-6-phosphate (T6P) in the induction of flowering in *Arabidopsis* [15–17]. Trehalose, a non-reducing disaccharide (composed of two glucose molecules) is present in many species, including bacteria, yeast, fungi, invertebrates, and plants, albeit in trace amounts. Enzyme *trehalose-6-phosphate synthase* (*TPS*) catalyzes the transfer of glucose from uridine diphosphate-glucose (UDPG) to glucose-6-phosphate (G6P), resulting in the formation of T6P. Subsequently, T6P is dephosphorylated by *trehalose phosphate phosphatase* (*TPP*) to produce trehalose [18, 19]. T6P functions as a critical signaling molecule in plants, relaying carbohydrate status to other signaling pathways and is often referred to as a "central hub in carbon signaling" [20]. It plays a vital role in various aspects of plant growth and development, including energy metabolism, stress responses, and floral transition, etc. It has also been implicated in the regulation of plant embryonic and vegetative development, flowering time regulation, meristem determinacy, and cell fate specification [21–24]. Although trehalose and trehalose-6-phosphate (T6P) are found only in trace levels in higher plants and have mostly lost their functions to sucrose, altering trehalose metabolism results in a number of different effects, such as reduced stress tolerance, changed leaf morphology, and embryo lethality, demonstrating that trehalose metabolites have crucial regulatory roles in plants metabolism [25, 26].

The role of T6P in flowering time regulation is well studied in model plant *Arabidopsis* [24, 27, 28]. The loss of *AtTPS1* activity causes *Arabidopsis* to flower extremely late even under inductive environmental conditions [24]. In *Arabidopsis*, T6P levels have been found to increase in response to changes in photoperiod, with higher levels promoting early flowering under long-day conditions. Conversely, under short-day conditions, T6P levels decrease, leading to delayed flowering [27]. Specifically, the T6P pathway operates within the photoperiod-inductive pathway, acting upstream of *FLOWERING LOCUS T* (*FT*) and *TWIN SISTER OF FT* (*TSF*) and is essential for the transition to flowering in the shoot apical meristem (SAM) and the induction of the florigen *FT* in leaves [24].

The *TPS* gene family has been identified in a wide range of plant species, including *Arabidopsis*, rice, and cotton [29–31]. The widespread presence of this gene family suggests that T6P signaling may be a common mechanism regulating flowering across different plant species. Similar to *Arabidopsis*, linseed is a facultative long day plant which flowers earlier under long days, however, eventually flowers under short days, albeit at a slower rate [32, 33].

In linseed, genotypic differences in photoperiod response have been reported [8, 34–36];, however, there are limited studies dissecting the regulation of flowering time in linseed [37–39]. Therefore, it is imperative to investigate the role of T6P in flowering time regulation in linseed. In the present study, the *TPS* gene family in linseed is identified and characterized, and its potential role in regulating flowering time is investigated.

## Materials and methods

### Identification and characterization of the linseed TPS gene family

Protein sequences of already identified *TPS* genes from diverse plant species, including *Arabidopsis thaliana* (11 sequences), *Oryza sativa* (11 sequences), *Gossypium spp.* (*G. arboreum*—14, *G. hirsutum*—24, *G. raimondii*—15), and *Solanum tuberosum* (8 sequences) were retrieved from the National Center for Biotechnology Information (NCBI) database. Further, the protein sequences of linseed were obtained from the Phytozome database (https://phytozome-next.jgi.doe.gov/). An HMM profile of *TPS* proteins was constructed using HMMER software [40] and employed to query the linseed protein sequences. The hits were screened for the presence of conserved domains using the NCBI batch CD-search tool (https://www.ncbi.nlm.nih.gov/Structure/bwrpsb/bwrpsb.cgi) [41]. Only the sequences possessing *TPS* gene family-specific conserved domains were considered as *TPS* genes. Subcellular localization of the linseed *TPS* genes was predicted using the WoLF PSORT tool (https://wolfpsort.hgc.jp) [42]. Physicochemical properties such as molecular weight, isoelectric point (pI), etc. were predicted using the ProtParam tool (https://web.expasy.org/protparam) [43], and the phosphorylation site prediction was performed using the ScanProsite tool (https://prosite.expasy.org/scanprosite) [44].

### Phylogenetic analysis and nomenclature

Multiple sequence alignment of the linseed *TPS* (*LuTPS*) protein sequences along with *Arabidopsis TPS* protein sequences was performed using t-coffee software version 13.41.0.28bdc39 [45]. A pairwise distance matrix between *Arabidopsis* and linseed *TPS* proteins was computed using MEGA 11 [46]. Each *LuTPS* gene was then named according to its closest *Arabidopsis* ortholog, as identified in the pairwise distance matrix. Subsequently, a phylogenetic tree was constructed employing the Maximum Likelihood (ML) method implemented in MEGA 11.

### Expression analysis of LuTPS genes

Expression analysis of *LuTPS* genes was conducted using the available transcriptome data of two early flowering-maturing germplasm accessions IC0523807 and IC0525939 (BioProject ID: PRJNA773597). The seed samples of these accessions were obtained from the National Genebank of India (NGB), Indian Council of Agricultural Research-National Bureau of Plant Genetic Resources (ICAR-NBPGR), New Delhi, India. The accessions conserved in NGB, India are accessible for research purposes following the standard material transfer agreement and as per the terms and conditions (https://nbpgr.org.in/nbpgr2023/germplasm-exchange-2/). The analysis included samples from the floral bud at two developmental stages, flower, leaf, and stem tissues. Normalized gene expression data, measured in transcripts per million (TPM), was used to examine the expression levels of *TPS* genes and plotted using ComplexHeatmap v2.10.0 [47] in the ‘*R*’ software environment. The expression-expression correlation between the LuTPS genes with major flowering genes and protein–protein interacting partners was analyzed using Pearson's correlation in the 'R' programming environment.

### Allele mining of LuTPS genes

Allele mining of LuTPS genes was carried out using whole genome resequencing data (paired-end short reads, Illumina NovaSeq platform) of two early (IC0523807, IC0525939) and two late-flowering accessions (EC0115148, EC0718827) (BioProject accession number: PRJNA1207411). The seed samples of the mentioned accessions were obtained from NGB, ICAR-NBPGR, New Delhi, India. The quality of sequencing reads was evaluated using FastQC tool [48]. Low-quality reads, along with adapter sequences and other contaminants, were removed using Trimmomatic tool v0.39 [49]. The cleaned reads were aligned to the reference genome using the ‘very-sensitive-local’ algorithm of Bowtie2 [50]. The resulting SAM files were converted to BAM format, and subsequently sorted, filtered, and indexed using SAMtools [51]. Reference-based SNP calling was performed against the linseed reference genome assembly ASM22429v2 (https://www.ncbi.nlm.nih.gov/datasets/genome/GCA_000224295.2/) [52] using the ‘mpileup’ function of the BCFtools [51]. A quality score threshold of ≥ 30 and a minimum read depth of ≥ 5 were applied for SNP variant calling.

### In-silico homology modeling and structural analysis

To assess the potential impact of amino acid substitutions on the three-dimensional (3D) structure of the TPS protein (LuTPS10.2), homology-based modeling was conducted using the SWISS-MODEL server (https://swissmodel.expasy.org/) [53]. For model generation, the template alpha, alpha-trehalose-phosphate synthase of *Glycine max* (UniProt id- I1N1F3; AlphaFold identifier, AF-I1N1F3-F1) was used which showed sequence identity of 76.89%, and 96% of the query coverage. The quality score of the generated models by the Global Model Quality Estimate (GMQE) was 0.831, which reflects high model reliability. Separate 3D models were constructed for the original protein (without amino acid substitutions) and another incorporating the specific amino acid substitution of interest. Potential energy of both the modeled protein structures was calculated using MutationExplorer tool (https://mutationexplorer.vda-group.de/mutation_explorer/) [54].

### Identification and enrichment of cis-regulatory elements

*Cis*-regulatory elements (*CRE*s) on 2 kb promoter sequences from the start codon of 18 *LuTPS* genes were identified, and their enrichments were studied by comparing their abundance against the 2 kb promoter sequences of 37,999 genes in the linseed genome to identify potential *CREs* that may influence the expression of *LuTPS* genes. The 2 kb promoter sequences starting from the start codon were extracted for all the annotated genes in the linseed genome (linseed reference genome assembly ASM22429v2 (https://www.ncbi.nlm.nih.gov/datasets/genome/GCA_000224295.2/) [52] using bedtools [55]. Promoter sequences shorter than 500 base pairs or containing more than 1,500 ‘NNNs’ were excluded from the analysis, resulting in a final set of 37,999 sequences for examination. Position weight matrix (PWM) data for 2,254 transcription factor binding sites (TFBSs) were downloaded from the Plant PAN 3.0 database (http://plantpan.itps.ncku.edu.tw/plantpan3/) [56] and used in conjunction with the PWMEnrich package in ‘*R*’ software to predict the distribution of *CREs* within the 2 kb promoter sequences of 37,999 linseed genes (background). The log-normal distribution of *CREs* within the 2 kb promoter sequences of the 18 *LuTPS* genes was then compared against the background distribution observed across the entire set of 37,999 linseed genes. A significance threshold of *q*-value ≤ 0.1 was applied to identify statistically significantly enriched *CREs* within the *LuTPS* gene promoters.

### Synteny analysis

A comparative synteny analysis of *LuTPS* genes was conducted with nine additional plant species representing diverse taxonomic groups comprising three oilseed crops (*Arabidopsis thaliana*, *Helianthus annuus*, *Sesamum indicum*), three cereal crops (*Hordeum vulgare*, *Oryza sativa*, *Triticum aestivum*), and three pulse crops (*Glycine max*, *Vigna radiata*, *Vigna unguiculata*). The complete set of annotated protein sequences (primary transcripts only) and corresponding GFF3 files specifying gene positions were downloaded from Phytozome for linseed and from NCBI for the other nine species. In instances where multiple transcript variants existed for a given gene, the longest transcript was used for the analyses. To identify potential orthologous gene pairs across species, pairwise BLAST analyses were performed for all possible combinations of the ten genomes, resulting in a total of 90 pairwise comparisons. Only the top five BLAST hits were considered for each gene. The co-localization of genes on chromosomes (synteny) across the ten plant species was studied using MCScanX software [57] with default settings (match score ≥ 50, match size ≥ 5 genes, gap penalty = −1, overlap window = 5, e-value ≤ 1e-05, and maximum gaps = 25). Subsequently, the synteny file generated by MCScanX was filtered to identify conserved syntenic blocks (CSBs) specifically encompassing the *LuTPS* genes. Genome-level synteny between linseed and the nine crops was visualized using SynVisio tool (https://synvisio.github.io/) [58], and the filtered CSBs (highlighting those containing *LuTPS* genes within genomic collinearity) were visualized using TBtools [59]. Gene collinearity networks (GCNs) for *LuTPS* genes were constructed with Cytoscape software [60].

### Protein–protein interaction

To understand protein–protein interacting partners of linseed TPS, the whole proteome of linseed (43,484 protein sequences) was uploaded to the STRING database and annotated. The interaction networks were generated based on the annotated proteome in the STRING database (https://version-12-0.string-db.org/organism/STRG0A33KPV) and the protein–protein interaction network of individual TPS was retrieved. The functional annotation of the interacting partners was performed using the PANNZER2 tool [61].

## Results

### Identification and in silico characterization of LuTPS gene family

**Fig. 1:**
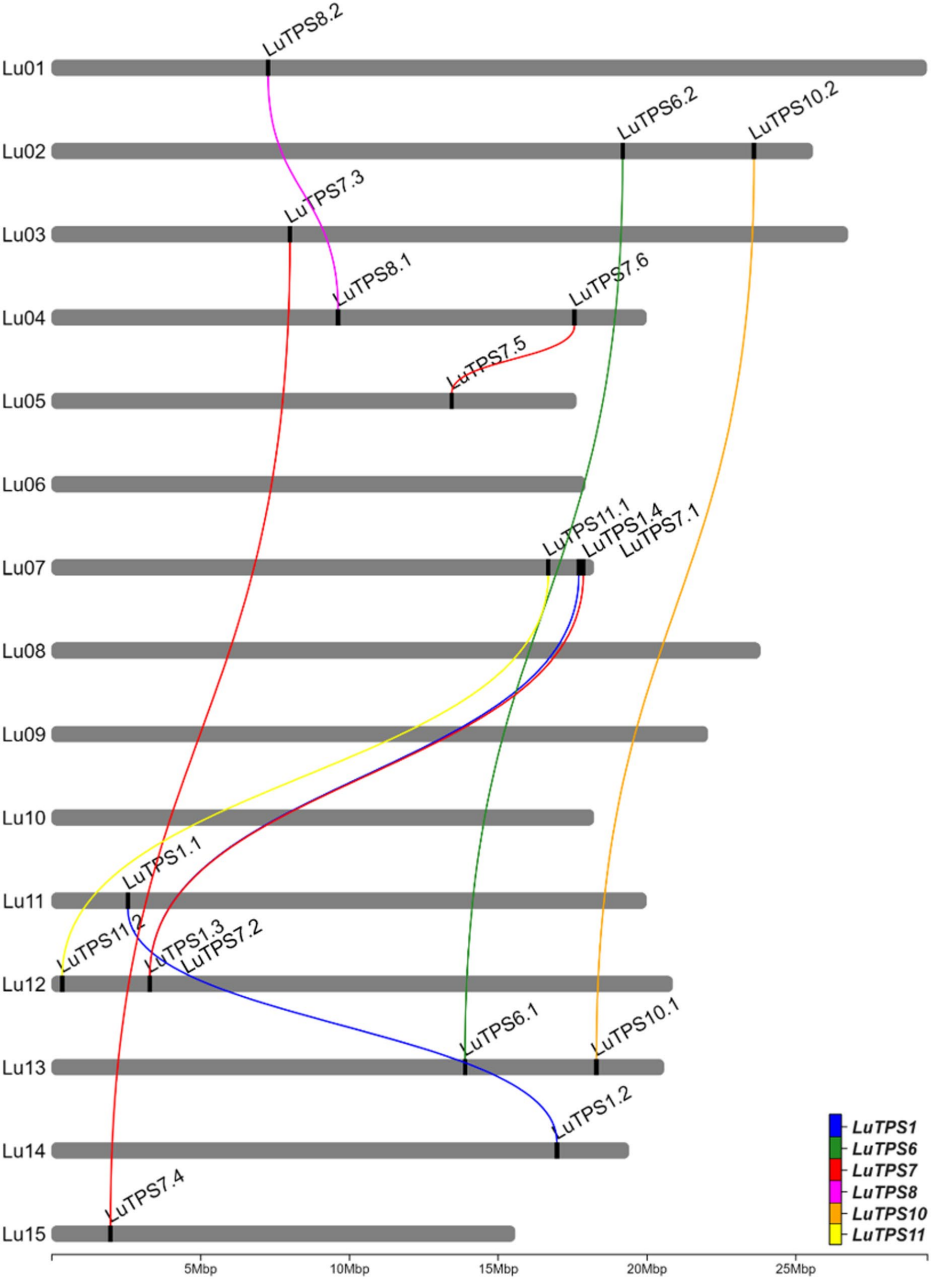
Chromosomal positions of trehalose-6-phosphate synthase genes in linseed and their paralogues. Lines connecting *TPS* genes indicate paralogous relation

A total of 18 *LuTPS* genes were identified in silico, distributed across 11 of the 15 linseed chromosomes, excluding Lu06, Lu08, Lu09, and Lu10 (Fig. 1, Table 1). The LuTPS proteins ranged from 800 (LuTPS11.2) to 971 (LuTPS1.1) amino acids long. The predicted isoelectric point (pI) values varied from 5.50 (LuTPS7.6) to 7.05 (LuTPS1.1), with an average pI of 6.08. The predicted localization of the LuTPS proteins varied across different cellular compartments, with the majority localized in the chloroplast (7 proteins), followed by cytoplasm (5 proteins), nucleus (5 proteins), and one in vacuole. The highest number of phosphorylation sites was predicted for LuTPS1.3 and LuTPS1.4 (47 each), followed by LuTPS6.1 and LuTPS6.2 (38 each), and LuTPS1.1 and LuTPS1.2 (34 each), whereas the lowest number of phosphorylation sites was found in LuTPS7.1 and LuTPS7.2 (23 each) (Table 1). The *LuTPS1* paralogs exhibited the most complex gene structures, with *LuTPS1.1* containing 16 exons and *LuTPS1.2*, *LuTPS1.3*, and *LuTPS1.4* each containing 17 exons. In contrast, other *LuTPS* genes displayed simpler structures, with exon numbers ranging from 2 (*LuTPS10.2*) to 4 (*LuTPS11.2*) (Figure S1).

**Table 1:** List of identified *TPS* genes in linseed and its *in-silico* characterization

Gene ID	Name	Arabidopsis ortholog	Subcellular localization	Protein length	Mol. weight (KDa)	pI	Phosphorylation sites				Amidation sites	ASN Glycosylation sites
							**CK2**	**PKC**	**TYR**	**CAMP**		
*Lus10013694*	*LuTPS1.1*	*At1g78580*	Chloroplast	971	109.26	7.05	16	16	0	2	3	5
*Lus10005559*	*LuTPS1.2*	*At1g78580*	Chloroplast	939	105.88	6.93	16	15	0	3	3	5
*Lus10015243*	*LuTPS1.3*	*At1g78580*	Chloroplast	946	106.37	6.51	26	19	0	2	2	6
*Lus10005412*	*LuTPS1.4*	*At1g78580*	Vacuole	957	107.96	6.69	26	19	0	2	3	6
*Lus10034585*	*LuTPS6.1*	*At1g68020*	Nucleus	855	97.18	5.65	18	16	1	3	1	1
*Lus10021805*	*LuTPS6.2*	*At1g68020*	Nucleus	855	97.14	5.60	16	18	1	3	1	1
*Lus10005425*	*LuTPS7.1*	*At1g06410*	Chloroplast	849	96.24	6.04	13	8	0	2	0	2
*Lus10015231*	*LuTPS7.2*	*At1g06410*	Chloroplast	849	96.17	6.10	13	8	0	2	0	2
*Lus10029258*	*LuTPS7.3*	*At1g06410*	Chloroplast	853	96.28	5.71	15	9	0	3	0	0
*Lus10007311*	*LuTPS7.4*	*At1g06410*	Chloroplast	853	96.44	5.76	15	9	0	3	0	1
*Lus10029821*	*LuTPS7.5*	*At1g06410*	Cytoplasm	838	95.19	5.53	18	7	0	1	1	3
*Lus10020741*	*LuTPS7.6*	*At1g06410*	Cytoplasm	838	95.23	5.50	19	6	0	1	1	4
*Lus10012990*	*LuTPS8.1*	*At1g70290*	Cytoplasm	865	98.12	6.01	12	10	1	1	1	2
*Lus10029175*	*LuTPS8.2*	*At1g70290*	Cytoplasm	865	97.95	6.11	12	11	0	1	0	2
*Lus10030853*	*LuTPS10.1*	*At1g60140*	Cytoplasm	864	97.74	5.90	13	10	0	2	1	2
*Lus10030635*	*LuTPS10.2*	*At1g60140*	Nucleus	897	101.33	5.90	11	12	0	1	1	2
*Lus10015509*	*LuTPS11.1*	*At2g18700*	Nucleus	850	96.36	6.25	15	11	1	1	0	0
*Lus10019982*	*LuTPS11.2*	*At2g18700*	Nucleus	800	90.75	6.13	15	10	1	1	0	0

### Phylogenetic analysis and nomenclature of linseed TPS

**Fig. 2:**
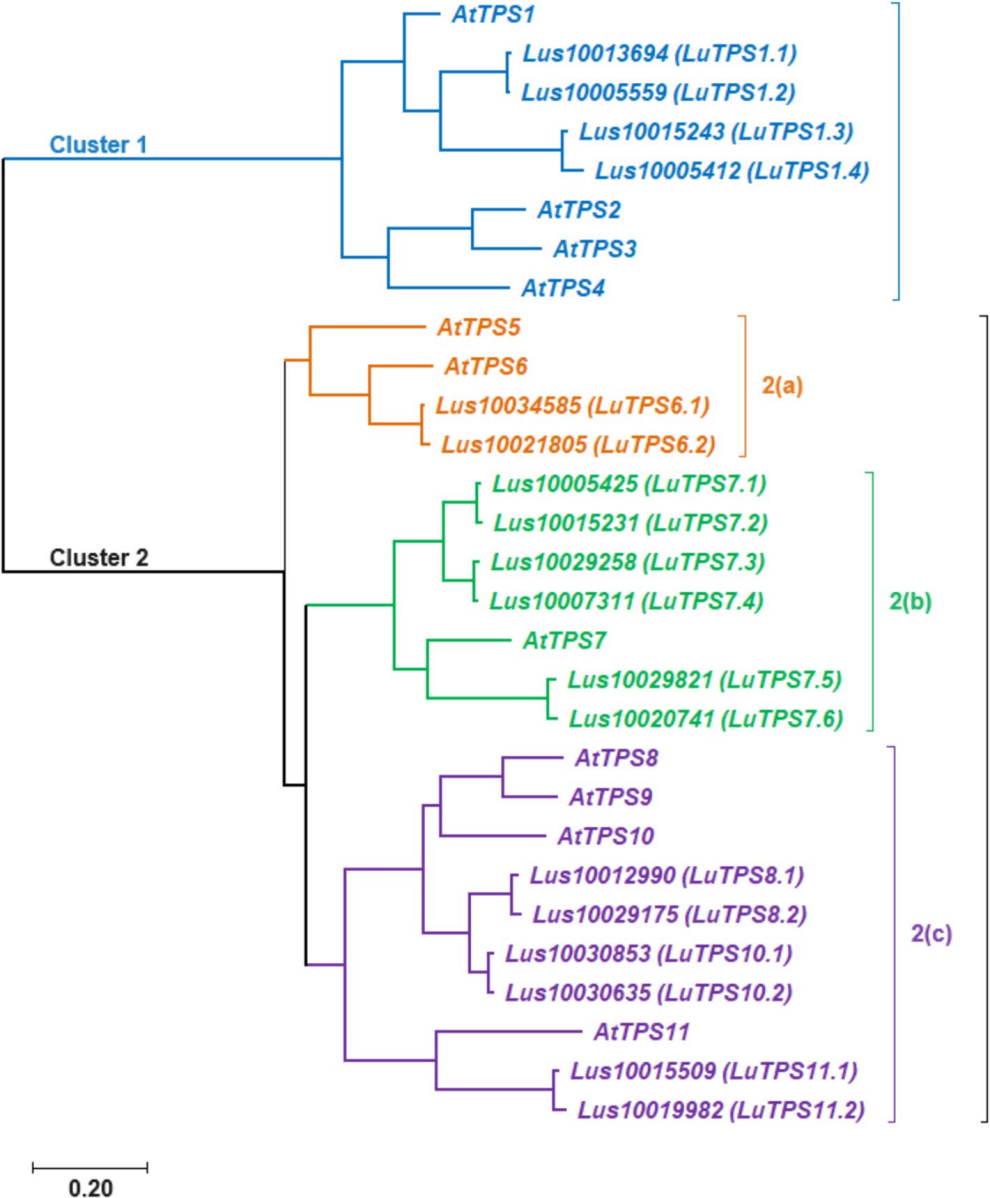
Phylogenetic analysis of trehalose-6-phosphate synthase (TPS) genes from linseed and *Arabidopsis thaliana*. The TPS genes are grouped into two major clusters, reflecting their evolutionary relationships

The *LuTPS* genes were named according to their closest *Arabidopsis* orthologs as identified in the pairwise distance matrix. In cases where multiple linseed genes showed similarity to the same *Arabidopsis* TPS, they were designated with numerical suffixes indicating their relative similarity to the *Arabidopsis* ortholog (Table 1). For phylogenetic analysis of LuTPS, the protein sequences of 18 LuTPS along with 11 AtTPS were aligned using t-coffee, and a phylogenetic tree was constructed using the ML method implemented in MEGA 11. The linseed TPS, along with *Arabidopsis* TPS, clustered into two distinct groups, Cluster 1 and Cluster 2 (Fig. 2). The LuTPS1 paralogues (LuTPS1.1, LuTPS1.2, LuTPS1.3, LuTPS1.4) were found in Cluster 1, alongside the AtTPS1. Cluster 1 also included AtTPS2, AtTPS3, and AtTPS4. Cluster 2 was further divided into three subclusters, 2a, 2b, and 2c. Subcluster 2a contained LuTPS6.1 and LuTPS6.2, along with AtTPS6, as well as AtTPS5. Subcluster 2b was exclusively composed of LuTPS7 (LuTPS7.1, LuTPS7.2, LuTPS7.3, LuTPS7.4, LuTPS7.5, *LuTPS7.6*) together with *AtTPS7*. Subcluster 2c included paralogues of LuTPS8 (LuTPS8.1, LuTPS8.2), LuTPS10 (LuTPS10.1, LuTPS10.2), and LuTPS11 (LuTPS11.1, LuTPS11.2), which clustered alongside AtTPS8, AtTPS10, AtTPS11, and AtTPS9.

### Expression analysis of *LuTPS* genes in vegetative and reproductive tissues

**Fig. 3:**
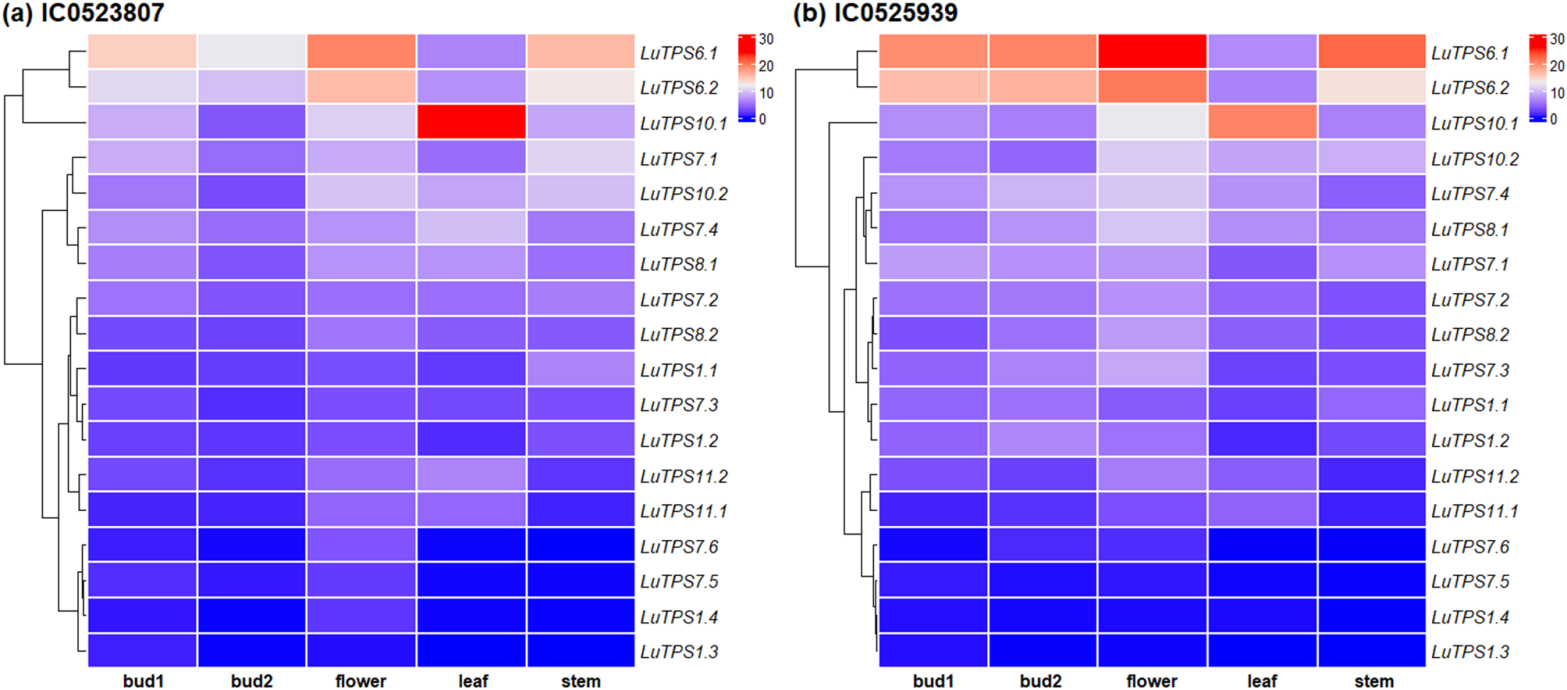
Gene expression profiles of *TPS* genes in reproductive and vegetative tissues of early-flowering linseed genotypes IC0523807 and IC0525939 based on transcriptome data. Gene names are displayed on the right, with expression-based hierarchical clustering shown on the left. The color gradient from red to blue represents transcript abundance in TPM (Transcripts Per Million), ranging from high to low expression levels

Gene expression profiles of the *LuTPS* genes in linseed were analyzed from the available transcriptome sequence data across four different tissues, bud at two developmental stages (bud1, bud2), flower, leaf, and stem, using RNA sequencing data from two early flowering-maturing accessions, IC0523807 and IC0525939. *LuTPS6.1*, *LuTPS6.2*, and *LuTPS10.1* showed relatively higher expression in all the studied tissues including floral buds, flowers, leaf and stem in both the accessions. *LuTPS10.1* showed conspicuously high expression in leaf in both the accessions (Fig. 3a, b). Most of the *LuTPS* genes except *LuTPS1.3*, *LuTPS1.4*, *LuTPS7.5*, and *LuTPS7.6* were found expressed in one or more studied tissue types in both the accessions. A few genes showed high expression across all tissues in both the early flowering accessions which included *LuTPS7.1*, *LuTPS7.2*, *LuTPS7.3*, *LuTPS7.4*, *LuTPS8.1*, *LuTPS8.2*, *LuTPS10.1*, and *LuTPS10.2*. In contrast, *LuTPS1.1*, *LuTPS1.2*, *LuTPS11.1*, and *LuTPS11.2* exhibited relatively higher expression in flower compared to other reproductive and vegetative tissues. Overall, the top most expressing LuTPS genes were *LuTPS6.1*, *LuTPS6.2*, and *LuTPS10.1.*

**Fig. 4:**
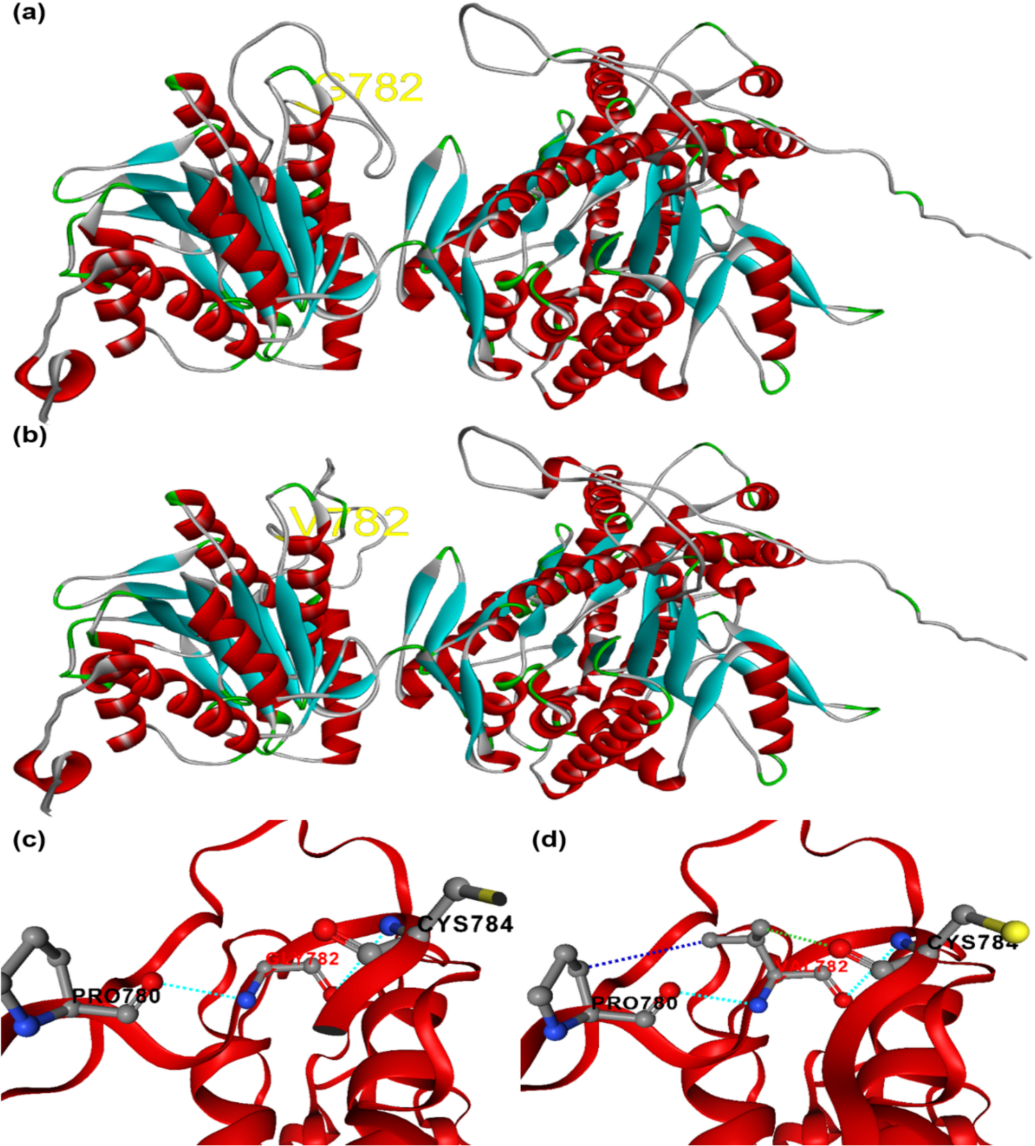
Homology-based 3D structures of the LuTPS10.2 protein variant in linseed, showing the amino acid substitution at position 782: (**a**) GLY-782 in late-flowering genotypes and (**b**) VAL-782 in early-flowering genotypes. Intramolecular interactions of the LuTPS10.2 variants are illustrated for (**c**) GLY-782 and (**d**) VAL-782

To further pinpoint the potential linseed TPS genes involved in flowering regulation linseed, TPS gene expressions was compared to that of gene expression of important flowering regulators including *FLOWERING LOCUS T (FT) (Lus10013532), FRUITFULL (FUL) paralogs (Lus10011349, Lus10021140), SUPPRESSOR OF OVEREXPRESSION OF CONSTANS 1 (SOC1) (Lus10036543), Squamosa Promoter Binding Protein-Like 9 (SPL9) (Lus10007984)* using the transcriptome of floral buds at two stages, flowers, leaves and stem of two early flowering linseed accessions IC0523807 and IC0525939 (Fig. 4)*.* A positive correlation of FT was observed only with LuTPS1.1; while *FUL (Lus10011349)* showed positive correlation with *LuTPS1.2, LuTPS6.1, LuTPS6.2, LuTPS7.2, LuTPS7.3, LuTPS7.4* and *LuTPS8.2,* indicating possible functional redundancy*.* Interestingly, *LuTPS10.1* was the only gene which showed positive correlation with expression of the other *FUL* paralog *(Lus10021140)* and *SOC1.* There was no significant correlation of any *TPS* gene expression to that of *SPL9,* indicating no probable role of the latter in regulating *TPS* gene expression.

### Allelic variation in LuTPS genes in early and late linseed accessions

To investigate the allelic variations in *LuTPS* gene family, available whole genome sequencing data of two early flowering-maturing (IC0523807, IC0525939) and two late flowering-maturing (EC0115148, EC0718827) linseed germplasm accessions (Bio-project ID-PRJNA1207411; Table S1) was used and the reference-based SNP calling was performed. Trait-specific SNPs (those capable of distinguishing between early and late flowering-maturing accessions) were identified in two genes, LuTPS*6.1* (3 SNPs: 2 SNPs in exons, 1 SNP in intron) (Table 2), and *LuTPS10.2* (3 SNPs, all in exons) (Table 3). Both the exonic SNPs in *LuTPS6.1* gene were synonymous in nature and therefore had no alteration in the protein sequence. Additionally, in the promoter sequence of the *LuTPS6.1* gene, a total of 16 SNPs/indels were identified (Table 2). However, these variations in the promoter region did not exhibit any clear pattern associated with early or late flowering phenotypes. In *LuTPS10.2*, from the 3 SNPs, one SNP was non-synonymous at nucleotide position 2439 (‘G’ in late flowering-maturing group changed to ‘T’ in early flowering-maturing group) which resulted in an amino acid substitution, Glycine (a non-polar amino acid) to Valine (an aliphatic and hydrophobic amino acid) at position 782 in the resulting protein (Table 3, Figure S2). The other two SNPs were synonymous, causing no change in the protein sequence. Further, in the promoter sequence of *LuTPS10.2*, a total of 9 SNPs, and 18 indels were identified (Table 3). Of these, 10 SNPs exhibited phenotype-specific patterns, differing between early and late flowering-maturing accessions. Further, two insertions of 2 and 11 nucleotides (at position −1117 to −1116 and −627 to −617, respectively) and a single nucleotide deletion (at position−685) were observed in both early flowering accessions.

**Table 2:** SNP haplotype of *LuTPS6.1* gene along with 2 kb promoter sequence in 2 early and 2 late flowering-maturing germplasm accessions of linseed. SNPs highlighted in bold font can differentiate between early and late flowering-maturing accessions

Nucleotide position	CDC Bethune (Reference)	Late flowering-maturing		Early flowering-maturing	
		**EC0115148**	**EC0718827**	**IC0523807**	**IC0525939**
−1903	G	G	G	A	G
−1812	C	T	C	T	T
−1529	A	A	-	A	A
−1528	C	C	-	C	C
−1484	G	G	A	G	G
−1289	A	A	-	A	A
−1288	T	T	-	T	T
−1287	A	A	-	A	A
−1286	A	A	-	A	A
−1285	A	A	-	A	A
−1284	A	A	-	A	A
−1283	A	A	-	A	A
−1253	A	G	A	G	G
−286	T	T	T	-	T
−285	C	C	C	-	C
−284	T	T	T	-	T
**1143**	**G**	**A**	**A**	**G**	**G**
**1716**	**G**	**T**	**T**	**G**	**G**
**2418**	**T**	**C**	**C**	**T**	**T**

**Table 3:** SNP haplotype of *LuTPS10.2* gene along with 2 kb promoter sequence in 2 early and 2 late flowering-maturing germplasm accessions of linseed. SNPs highlighted in bold font can differentiate between early and late flowering-maturing accessions

Nucleotide position	CDC Bethune (Reference)	Late flowering-maturing		Early flowering-maturing	
		**EC0115148**	**EC0718827**	**IC0523807**	**IC0525939**
**−1989**	**C**	**C**	**C**	**G**	**G**
**−1974**	**C**	**C**	**C**	**T**	**T**
**−1960**	**C**	**C**	**C**	**T**	**T**
**−1951**	**G**	**G**	**G**	**A**	**A**
**−1886**	**G**	**G**	**G**	**A**	**A**
**−1878**	**T**	**T**	**T**	**C**	**C**
**−1796**	**G**	**G**	**G**	**A**	**A**
−1735	C	C	T	C	C
−1458	T	T	T	T	-
−1457	A	A	A	A	-
−1456	A	A	A	A	-
−1455	A	A	A	A	-
**−1117**	**-**	**-**	**-**	**C**	**C**
**−1116**	**-**	**-**	**-**	**T**	**T**
−984	C	C	C	T	C
**−685**	**T**	**T**	**T**	**-**	**-**
**−627**	**-**	**-**	**-**	**C**	**C**
**−626**	**-**	**-**	**-**	**A**	**A**
**−625**	**-**	**-**	**-**	**A**	**A**
**−624**	**-**	**-**	**-**	**T**	**T**
**−623**	**-**	**-**	**-**	**G**	**G**
**−622**	**-**	**-**	**-**	**T**	**T**
**−621**	**-**	**-**	**-**	**G**	**G**
**−620**	**-**	**-**	**-**	**A**	**A**
**−619**	**-**	**-**	**-**	**G**	**G**
**−618**	**-**	**-**	**-**	**T**	**T**
**−617**	**-**	**-**	**-**	**T**	**T**
**1063**	**C**	**C**	**C**	**T**	**T**
**2439**	**G**	**G**	**G**	**T**	**T**
**2624**	**A**	**A**	**A**	**G**	**G**

### Effect of amino acid substitution on 3D structure of TPS protein

**Fig. 5:**
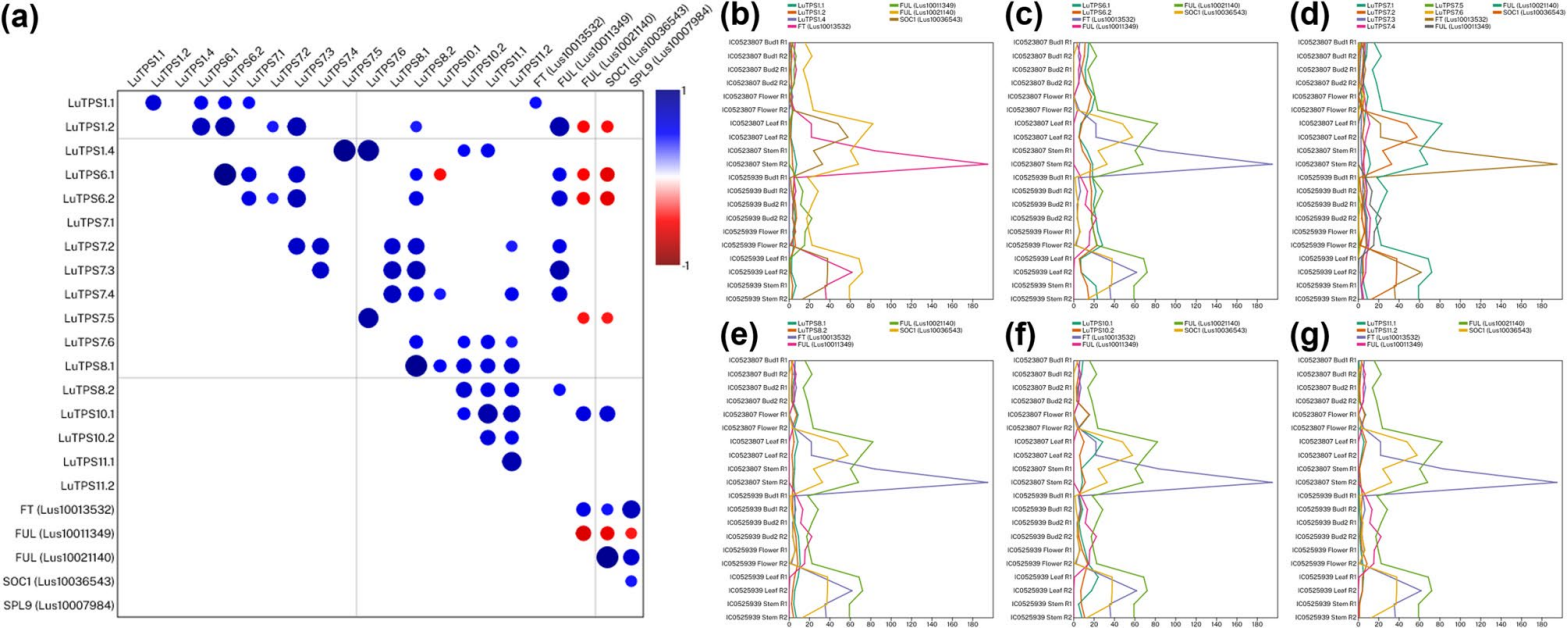
Correlation of expression of *TPS* genes and key flowering genes *FT (Lus10013532), FUL paralogs (Lus10011349, Lus10021140), SOC1 (Lus10036543)* and *SPL9 (Lus10007984)* in linseed*.* **a** Pairwise correlation between gene pairs. Color gradient of the circle from blue to red denotes positive to negative correlation. Size of the circle indicates the strength of p value. **b**, **g** Line plots depicting the normalized expression (Transcripts per Million) of *TPS* paralogs and flowering genes across tissues, floral bud 1, bud 2, flower, leaves, and stem in early-flowering linseed accessions IC0525939 and IC0523807. Expression data under NCBI, BioProject ID PRJNA773597 was used

To evaluate the impact of the amino acid substitution on the LuTPS10.2 protein's 3D structure, homology-based modeling was performed and the 3D structures of both the original protein (prior to amino acid substitution) and the modified protein (after substitution) were predicted and compared to evaluate any structural changes (Fig. 5a-d). Notably, the proteins from the early flowering-maturing group demonstrated an increase in intramolecular interactions, which typically enhances protein stability (Fig. 5c, d). The substitution of ‘G’ with ‘V’ in the early flowering-maturing group led to a significant reduction in potential energy, contributing to a stabilizing effect on the proteins within these accessions (Table 4).

**Table 4:** Potential energy of *LuTPS10.2* protein before and after amino acid substitution in late and early flowering-maturing accessions

Accession	Potential energy (kcal/mol)	Accession	Potential energy (kcal/mol)	Difference (kcal/mol)	Effect of mutation
EC0115148, EC0718827	−708.44	IC0523807, IC0525939	−715.47	−7.03	Stabilizing

### Analysis of CREs in LuTPS genes and their enrichment

2 kb promoter sequences upstream of the start codon of 37,999 linseed genes were extracted from the linseed genome assembly. The position weight matrix data of 2,254 TF binding sites (TFBS) from the PlantPAN 3.0 database were used to predict the occurrence of *CRE* motifs within these promoter sequences. The *CREs* within the 2 kb promoter regions of 18 LuTPS genes were identified, and their enrichment was assessed by statistically comparing their frequency against the background frequency across the entire linseed genome (37,999 genes). A total of 32 *CREs* were identified as significantly enriched in the promoter sequences of *LuTPS* genes compared to the average genomic distribution at a threshold of *q-*value ≤ 0.1 (Table 5). Among the significantly enriched *CREs*, flowering and photoperiod related *CREs* included *TF_motif_seq_0250*, *TF_motif_seq_0146*, *TF_motif_seq_0321*, *TFmatrixID_1221*, *TFmatrixID_0797*, and *TF_motif_seq_0481*. It is intriguing to note that from the 32 enriched *CRE*s, at least 15 were related to Dof-type domain-containing protein (Table 5). In addition, the promoter sequences of individual *LuTPS* genes were also analyzed for the presence of *CRE*s using the PlantPAN4 database [62]. The analysis identified a total of 104 *CRE*s, each present at least once in the promoter region of every *LuTPS* gene (Table S3). It is also important to highlight that six of the enriched *CRE*s (*TF_motif_seq_0250, TF_motif_seq_0315, TF_motif_seq_0344, TF_motif_seq_0238, TF_motif_seq_0321,* and *TF_motif_seq_0458*) were consistently present in the promoter of all TPS genes in linseed (Table 5, Table S3).

**Table 5:**
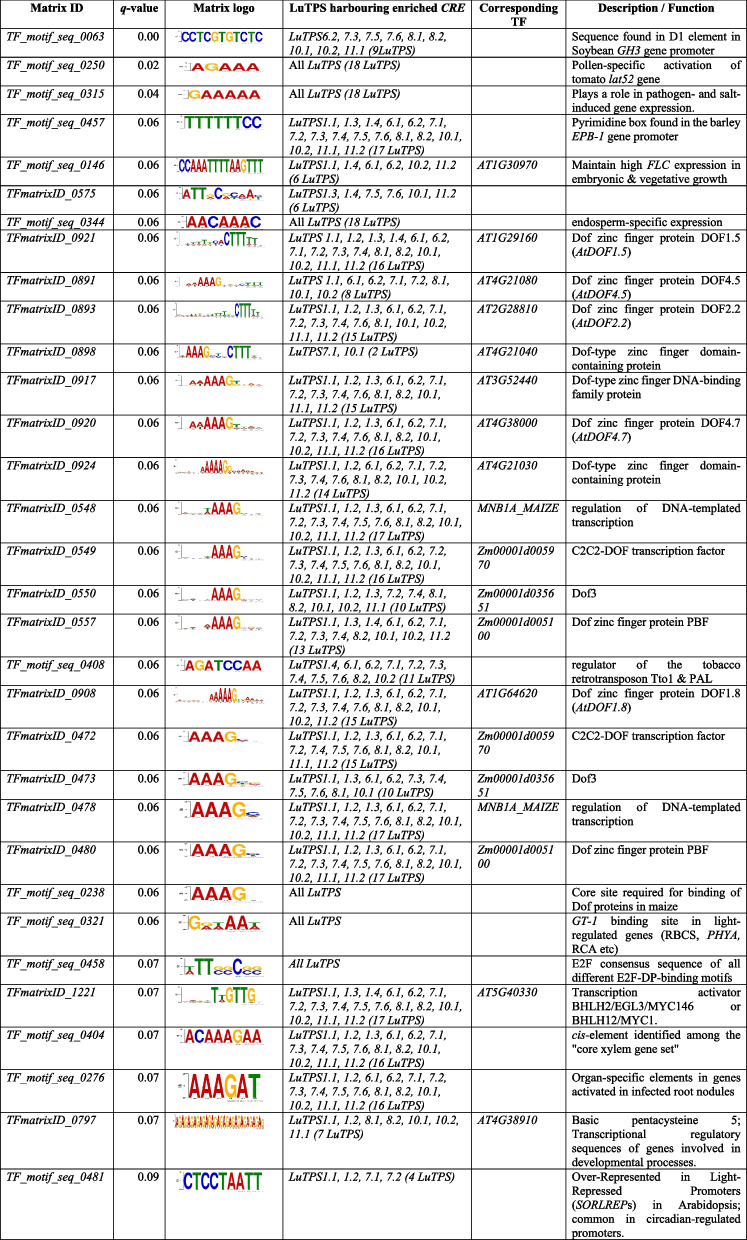
List of *cis*-regulatory elements enriched in *LuTPS* promoter sequences

### Genome scale syntenic network analysis of linseed and nine other plant genomes

**Fig. 6:**
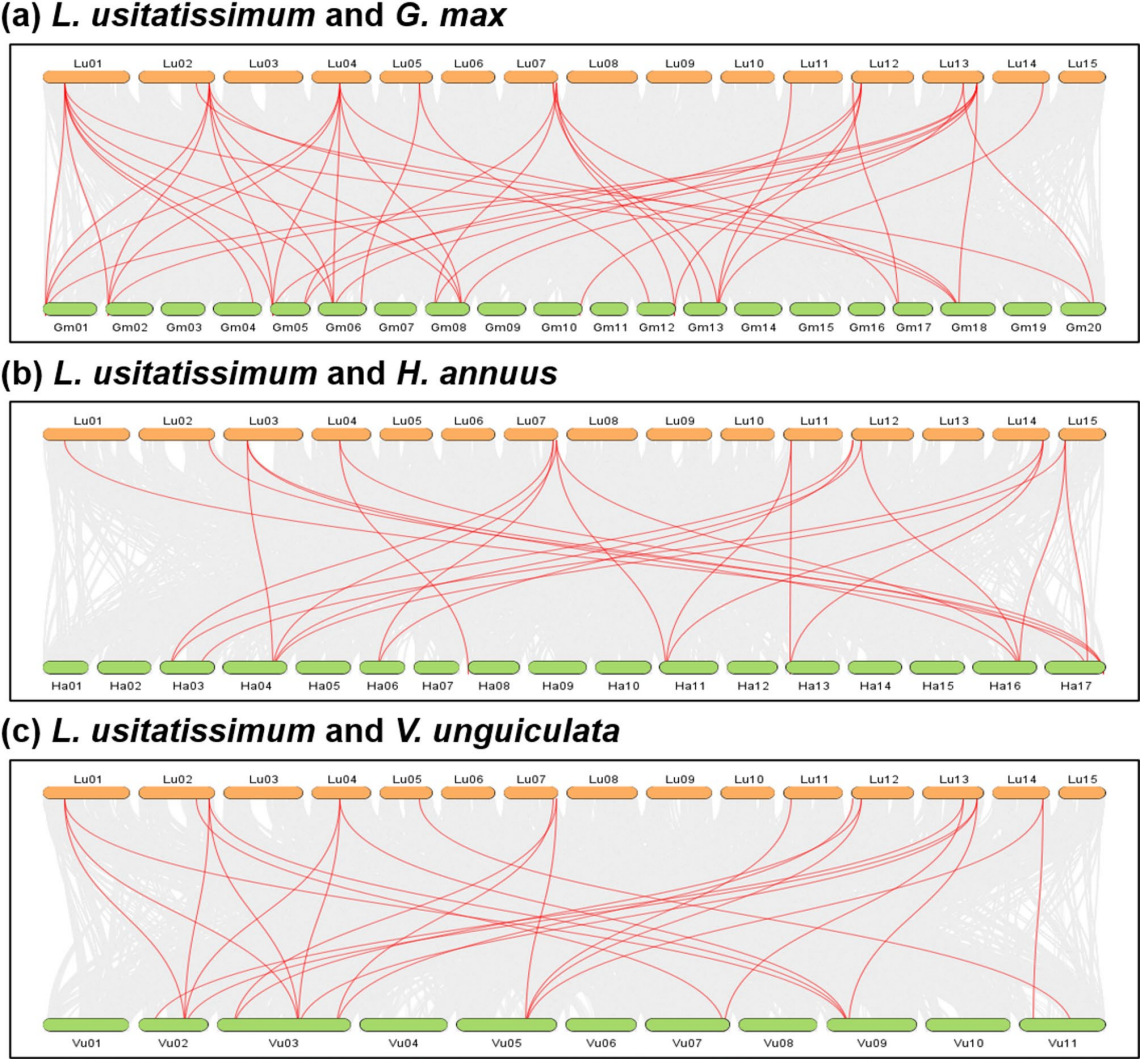
Genome-wide synteny analysis of linseed with soybean (*Glycine max*) (**a**), sunflower (*Helianthus annuus*) (**b**), and cowpea (*Vigna unguiculata*) (**c**). The genome wide conserved syntenic blocks (CSB) between the two species are depicted in grey shade, and the CSBs harbouring linseed TPS are shown with red lines

To understand synteny of TPS genes in different crop plants, The genome scale syntenic network analysis of linseed and nine other plants representing cereals, oilseeds, pulses, and a model plant species (*Arabidopsis*, rice, barley, wheat, sesame, sunflower, soybean, greengram, and cowpea) was performed. A total of 68,930 conserved syntenic blocks (CSBs) were identified in the studied 10 plant species (Table 6). Among the comparisons, the highest number of CSBs involving linseed was found with soybean, (3,673 CSBs), followed by sunflower (2,159), cowpea (2,092), and sesame (2,018) while barley exhibited the fewest CSBs with linseed (588) (Fig. 6, Figure S3, Table 6). Notably, 179 of the 68,930 CSBs contained at least one *LuTPS* gene (Table 7). The highest number of *LuTPS*-containing CSBs was observed between linseed and soybean (43), followed by cowpea (25), sunflower (24), and sesame (22). Linseed itself had 15 intraspecific CSBs with gene counts per CSB ranging from 9 to 399 (Fig. 6, Table 7).

**Table 6:** Number of total CSBs identified among the 10 crops under study. The numbers in parentheses indicate the size (number of genes) of the smallest and largest CSBs. The numbers in curly braces denote the count of CSBs in the plus and minus orientations, respectively

	**At**	**Gm**	**Ha**	**Hv**	**Lu**	**Os**	**Si**	**Ta**	**Vr**	**Vu**
At	231 (6—196) {102/129}	2443 (5—94) {1214/1229}	1434 (6—56) {721/713}	312 (6—21) {161/151}	1968 (6—74) {1019/949}	513 (6—22) {253/260}	1252 (6—120) {650/602}	907 (6—21) {472/435}	1162 (6—84) {558/604}	1305 (6—99) {661/644}
Gm		1038 (6—939) {529/509}	2859 (5—131) {1414/1445}	1128 (6—26) {563/565}	3673 (5—107) {1848/1825}	1567 (6—29) {772/795}	2368 (6—211) {1219/1149}	3188 (6—26) {1581/1607}	1776 (5—690) {916/860}	1715 (5—1127) {886/829}
Ha			720 (6—151) {390/330}	318 (6—22) {164/154}	2159 (6—64) {1116/1043}	524 (6—25) {288/236}	1607 (6—126) {847/760}	883 (6—24) {483/400}	1380 (6—87) {674/706}	1552 (6—125) {792/760}
Hv				112 (6—98) {58/54}	588 (6—35) {302/286}	525 (5—873) {265/260}	655 (6—49) {354/301}	1382 (5—2585) {707/675}	501 (6—38) {255/246}	638 (6—36) {343/295}
Lu					811 (5—916) {421/390}	883 (6—35) {424/459}	2018 (6—133) {1042/976}	1662 (6—38) {798/864}	1798 (5—116) {908/890}	2092 (6—118) {1065/1027}
Os						193 (6—437) {111/82}	900 (6—52) {470/430}	1629 (5—842) {842/787}	679 (6—40) {352/327}	874 (6—44) {427/447}
Si							287 (6—152) {149/138}	1850 (6—48) {1010/840}	1129 (5—153) {569/560}	1237 (6—176) {618/619}
Ta								1853 (5—2878) {994/859}	1398 (6—34) {684/714}	1789 (6—34) {901/888}
Vr									261 (6—141) {131/130}	863 (5—788) {425/438}
Vu										341 (6—247) {170/171}

At: *Arabidopsis*, Gm: Soybean, Ha: Sunflower, Hv: Barley, Lu: Linseed, Os: Rice, Si: Sesame, Ta: Wheat, Vr: Greengram, Vu: Cowpea

**Table 7:** Number of CSBs containing at least one *LuTPS* gene. The numbers in parentheses indicate the size (number of genes) of the smallest and largest CSBs. The numbers in curly braces denote the count of CSBs in the plus and minus orientations, respectively

	**At**	**Gm**	**Ha**	**Hv**	**Lu**	**Os**	**Si**	**Ta**	**Vr**	**Vu**
Lu	19 (7—39) {10/9}	43 (6—57) {19/24}	24 (6—63) {12/12}	5 (7—9) {3/2}	15 (9—399) {6/9}	9 (6—10) {3/6}	22 (6—66) {11/11}	6 (6—9) {3/3}	11 (7—18) {5/6}	25 (6—57) {13/12}

At *Arabidopsis*, Gm Soybean, Ha: Sunflower, Hv: Barley, Lu: Linseed, Os: Rice, Si: Sesame, Ta: Wheat, Vr: Greengram, Vu: Cowpea

### Syntenic gene collinearity networks (GCN) of linseed TPS

**Fig. 7:**
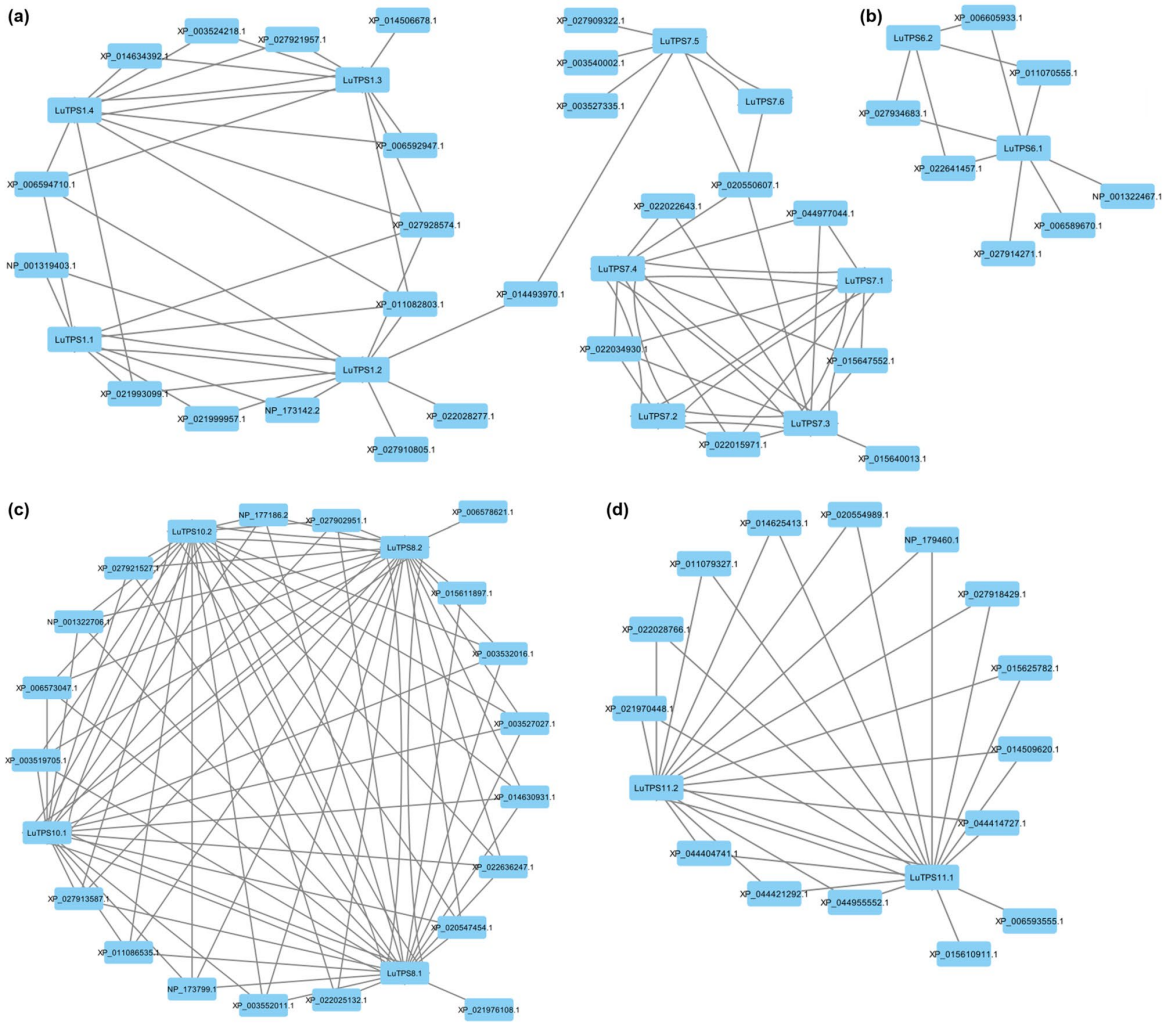
Gene collinearity networks (GCNs) derived from conserved syntenic blocks (CSBs) containing TPS genes. Four GCN clusters (I–IV) are shown in panels (**a**–**d**). Each node represents a gene, annotated with the corresponding CSB, and edges indicate syntenic relationships between genes

In order to identify *LuTPS*-specific syntenic block networks (SBN), the 179 CSBs (containing at least one *LuTPS* gene) were analyzed using Cytoscape software [48]. Accordingly, the 179 CSBs clustered into four distinct SBNs. The interaction of linseed *TPS* genes within these four SBNs was visualized as nodes (representing genes) and edges (representing syntenic relationships). Consequently, the linseed *TPS* genes formed four gene collinearity networks (GCN) (Fig. 7). Each node (gene) within the GCN represents the CSB in which this gene was located, while the edges highlight the syntenic relationships between them. The largest cluster, GCN Cluster-I (Fig. 7a), comprised of 35 genes, including 10 linseed *TPS* genes, *LuTPS1.1, LuTPS1.2, LuTPS1.3, LuTPS1.4, LuTPS7.1, LuTPS7.2, LuTPS7.3, LuTPS7.4, LuTPS7.5,* and *LuTPS7.6*. The remaining genes in this GCN were *TPS* genes from soybean, sunflower, cowpea, sesame, *Arabidopsis*, greengram, barley, and rice. The highest syntenic relationship for linseed *TPS* genes was observed with soybean and sunflower (6 genes each), followed by cowpea (4 genes), sesame (2 genes), *Arabidopsis* (2 genes), greengram (2 genes), rice (2 genes), and barley (1 gene). Within Cluster-I, subcluster-Ia consists of four linseed *TPS genes (LuTPS1.1, LuTPS1.2, LuTPS1.3*, and *LuTPS1.4*). Syntenic relationships were observed between *LuTPS1.1 & LuTPS1.2,* and between *LuTPS1.3 & LuTPS1.4,* though no direct connections were found between the two pairs. However, connections were observed with *TPS* genes from other plants, suggesting an ancient duplication event that led to the divergence of these gene pairs. Subcluster-Ia and subcluster-Ib were connected through a syntenic relationship between *LuTPS1.2* and *LuTPS7.5*, facilitated by a *TPS* gene from greengram (XP_014493970.1). In subcluster-Ib, two linseed *TPS* genes*, LuTPS7.5* and *LuTPS7.6*, displayed direct syntenic relationships. *LuTPS7.5* also showed syntenic connections with *TPS* genes from cowpea and soybean. Subcluster-Ib was linked to Subcluster-Ic through syntenic relationships involving *LuTPS7.5* and *LuTPS7.6*, both of which exhibited synteny with a *TPS* gene from sesame (XP_020550607.1). Subcluster-Ic comprised four linseed TPS genes (*LuTPS7.1, LuTPS7.2, LuTPS7.3,* and *LuTPS7.4*), all of which exhibited direct syntenic relationships with each other, indicating a high degree of conservation within this group. Cluster II was the smallest, with only 9 genes, including two linseed *TPS* genes, *LuTPS6.1* and *LuTPS6.2*, which did not share direct syntenic interactions (Fig. 7b). Other genes in this cluster were from soybean, cowpea, greengram, sesame, and an *Arabidopsis* UDP-Glycosyltransferase/trehalose-phosphatase family protein (NP_001322467.1). *LuTPS6.1* displayed direct syntenic connections with 7 genes, including the *Arabidopsis* gene, whereas *LuTPS6.2* was connected with TPS genes from soybean, cowpea, greengram, and sesame. Interestingly, this cluster appears specific to dicot species, as no *TPS* genes from monocots (rice, barley, and wheat) were represented. Cluster III, containing 23 genes, featured four linseed TPS genes, *LuTPS8.1, LuTPS8.2, LuTPS10.1,* and *LuTPS10.2* displaying direct syntenic relationships with one another (Fig. 7c). Other genes in the cluster are from soybean, cowpea, *Arabidopsis*, sunflower, sesame, greengram, and rice. Notably, wheat and barley *TPS* genes are absent from this cluster. *LuTPS8.1* and *LuTPS8.2* both interacted with 18 other TPS genes. The syntenic relationship with the sunflower TPS gene (XP_021976108.1) was specific to LuTPS8.1, while LuTPS8.2 uniquely showed interaction with the soybean TPS gene (XP_006578621.1). Additionally, both *LuTPS10.1* and *LuTPS10.2* demonstrate syntenic connections with 17 other TPS genes. Cluster IV consists of 17 genes, including two linseed *TPS* genes, *LuTPS11.1* and *LuTPS11.2*, alongside TPS genes from other species (Fig. 7d). Notably, LuTPS11.1 and LuTPS11.2 exhibited a direct syntenic relationship with each other. Additionally, LuTPS11.1 displayed syntenic connections with all 16 other genes in the cluster, whereas LuTPS11.2 was syntenically linked to 14 genes, with the exceptions being the TPS genes from rice (XP_015610911.1) and soybean (XP_006593555.1). Notably, this cluster included *TPS* genes from all the ten plant species under study.

**Fig. 8:**
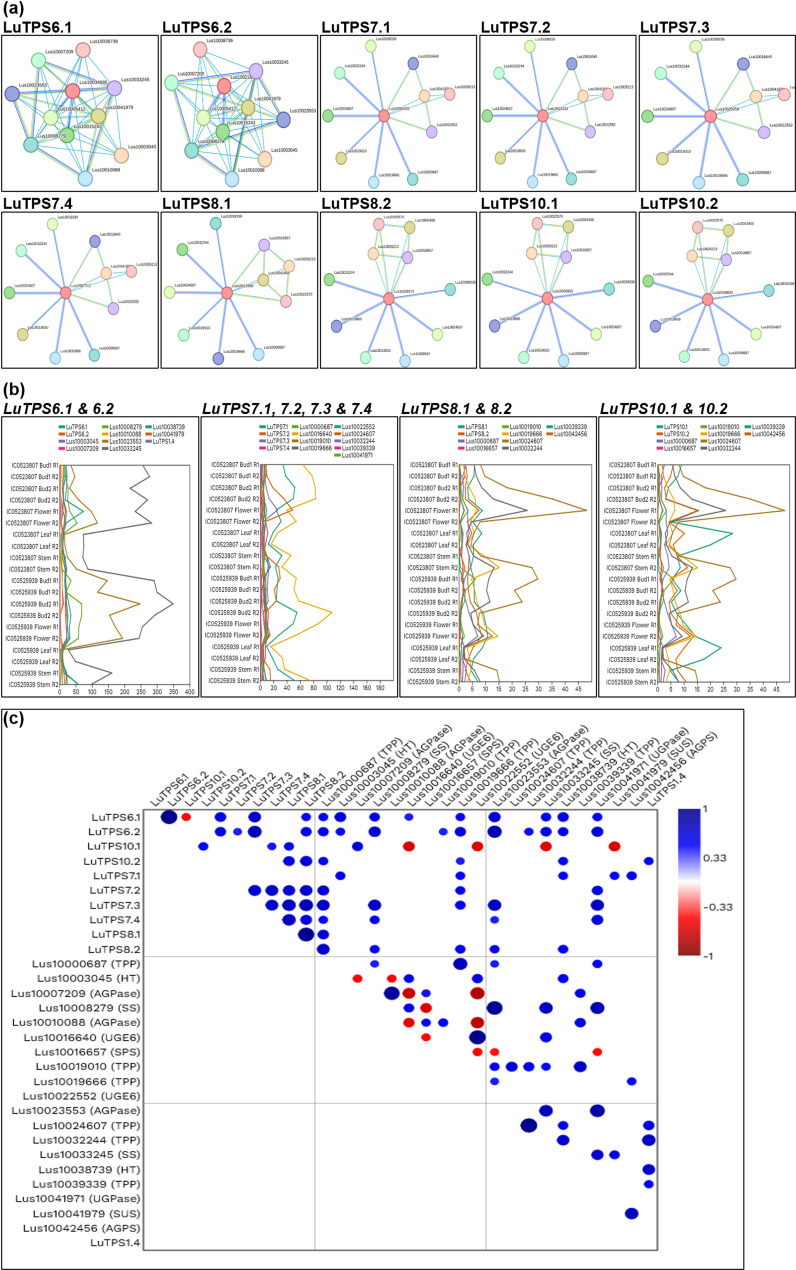
LuTPS protein–protein interactions and co-expression with the potential interacting genes. **a** Protein–protein interaction network of linseed TPS as identified using string database. The central node with red color is TPS protein, other nodes with different colors indicate the interacting protein and edges indicate their interactions. **b** Co-expression of *TPS* genes and respective potential interacting partners in floral buds at two developmental stages, flower, leaf and stem in two biological replicates of early flowering linseed genotypes IC0523807 and IC0525939 based on transcriptome data. The gene expression value is in TPM. **c** Correlation of gene expression of linseed *TPS* genes and their potential interacting partners. Size of the circle indicates the strength of p value and color gradient of the circle from blue to red denotes positive to negative correlation

To study if any of the genes in the linseed TPS specific CSBs also show any molecular interactions with TPS, we studied protein–protein interaction (PPI) network of TPS using the STRING database. The potentially interacting partners of each linseed TPS have been given in Table S4. There were a total of 27 unique interacting proteins identified for all 18 linseed TPS. Most linseed TPS paralogues shared the same interacting partners. Three of the interacting proteins, Lus10017984 (Uncharacterized protein), Lus10038739 (Hexosyltransferase), and Lus10041979 (Sucrose synthase) were part of the linseed TPS specific CSBs. Of which, Lus10017984 (Uncharacterized protein) showed PPI with LuTPS1.1, LuTPS1.2, LuTPS11.2. The other proteins, Lus10041979 (Sucrose synthase) showed PPI with 6 linseed TPS (LuTPS1.1, LuTPS1.2, LuTPS1.3, LuTPS1.4, LuTPS6.1, LuTPS6.2), whereas Lus10038739 (Hexosyltransferase) showed PPI specifically with LuTPS6.1, LuTPS6.2 (Table S4, Fig. 8a). From the 18 LuTPS, for the top ten expressing TPS genes (LuTPS6.1, LuTPS6.2, LuTPS10.1, LuTPS10.2, LuTPS7.1, LuTPS7.2, LuTPS7.3, LuTPS7.4, LuTPS8.1, LuTPS8.2) (Fig. 3), PPI network was drawn (Fig. 8a). For these 10 TPS, there were 20 unique interacting proteins, consisting mainly, trehalose 6-phosphate phosphatases, glucose-1-phosphate adenylyltransferase, sucrose-phosphate synthase, hexosyltransferase etc. (Table S5). The co-expression analysis of these TPS genes with the interacting partners was done using the transcriptome data of two early flowering linseed accessions in floral buds at two stages, flowers, leaves and stem (Fig. 8b). Correlation analysis of *LuTPS* and their respective interacting partners showed significant positive correlation of Lus10038739 (Hexosyltransferase) with six TPS genes, LuTPS6.1, LuTPS6.2, LuTPS10.2, LuTPS7.1, LuTPS7.2, and LuTPS8.2 (Fig. 8c). Interestingly, Lus10041979 (Sucrose synthase) showed significant positive and negative correlation with LuTPS7.1 and LuTPS10.1, respectively. Five of the ten potential interacting partners of LuTPS6.1 and LuTPS6.2 showed positive correlation with them, which included hexosyltransferases (Lus10038739, Lus10003045), starch synthases (Lus1003324, Lus10008279), and glucose-1-phosphate adenylyltransferase (Lus10023553). It is interesting to note that all linseed TPS, except LuTPS6.1 and LuTPS6.2 showed at least one of the interacting partners as trehalose 6-phosphate phosphatase.

## Discussion

### The TPS gene family in linseed

In the present study, a comprehensive in silico analysis identified 18 *TPS* genes in the linseed genome, distributed across 11 of its 15 chromosomes (Table 1). Among diploid plant species where the *TPS* gene family has been identified, linseed with 18 *TPS* genes represents one of the largest *TPS* families. *TPS* gene families in other plants such as *Arabidopsis thaliana*, *Brassica oleracea*, *Brassica rapa*, *Oryza sativa*, and *Solanum lycopersicum* consist of 11, 12, 15, 11, and 11 genes, respectively [30, 31, 62–64]. However, as expected, tetraploid species such as *Brassica napus* and *Gossypium hirsutum* exhibit relatively larger *TPS* families, with 30 and 53 genes, respectively [29, 31].

Identification of multiple paralogous genes in linseed suggests a potential functional diversity within this group, as these genes are linked to key regulatory processes, including stress response and carbohydrate metabolism. In *Arabidopsis*, *TPS1* plays a critical role in T6P synthesis, which is a central regulator of carbon allocation and flowering [24]. The multiple orthologs (four *TPS1*, two *TPS6*, six *TPS7*, two *TPS8*, two *TPS10*, and two *TPS11*) identified in linseed suggests a potentially expanded functional role, possibly associated with the plant's response to environmental stress, growth regulation, and development. Further, the presence of multiple paralogous genes in linseed (2 to 6) may point to redundancy or sub-functionalization, where different paralogs perform distinct roles depending on the tissue type or environmental conditions. Notably, *TPS* genes corresponding to *Arabidopsis TPS2*, *TPS3*, *TPS4*, *TPS5*, and *TPS9* were absent in linseed. The absence of these genes could be an indicator of either lineage-specific gene loss during evolution or a shift in functional requirements in linseed compared to *Arabidopsis*. This absence might also reflect different evolutionary pressures, where certain trehalose biosynthetic pathways or related regulatory processes have become less essential for the survival and adaptation of linseed in its environmental niche.

### Duplication of the LuTPS gene family

Sequence similarity analysis of the *LuTPS* gene family suggests that the family has undergone one or more duplication events, leading to the expansion of this gene family. The 18 identified *LuTPS* genes probably originated from the duplication of an ancestral set of *LuTPS* genes. Each *LuTPS* gene demonstrates a high degree of sequence similarity with its corresponding paralog (Fig. 1, Table S2). This can be attributed to whole-genome duplication (WGD) events in linseed. Linseed was reported to have undergone two major WGD events, a more recent event estimated to have occurred between 3.7 and 6.8 million years ago, and an ancient event between 23.8 and 44.1 million years ago [52]. The recent WGD event appears to have driven the expansion of the *LuTPS* gene family, resulting in the 18 identified genes likely derived from the duplication of ancestral *TPS* genes.

Gene duplication is a well-documented evolutionary mechanism that plays a crucial role in the formation of gene families. This process allows paralogous genes to either retain their original functions, diverge into new functional roles, or adapt to unique regulatory pathways. The high sequence identity observed between *LuTPS* paralogs indicates that these genes may still perform similar biological functions. However, subtle differences in their regulatory mechanisms or functional specializations may have evolved post-duplication, allowing for the fine-tuning of trehalose-related processes in linseed. The observed duplication events also underscore the importance of *TPS* genes in the evolutionary adaptation of linseed, potentially offering enhanced flexibility for responding to environmental stresses such as drought, salinity, or extreme temperature.

### Gene expression analysis of TPS genes in linseed

Expression analysis of the *LuTPS* gene family in two early flowering-maturing linseed accessions, IC0523807 and IC0525939, reveals distinct tissue-specific expression patterns. In both accessions, several *LuTPS* genes showed heightened expression in reproductive tissues, indicating their critical involvement in flowering and reproductive development. For example, *LuTPS6.1* and *LuTPS6.2* consistently exhibited high expression across all tissues, with particularly elevated levels in the flower, bud1, and bud2 stages (Fig. 3a, 3b). This indicates an involvement of these genes in reproductive processes, likely influencing the regulation of flowering by enhancing the expression of key floral regulators florigen *FT* and *TSF*. It is well established that the T6P pathway functions upstream of *FT* in the photoperiodic flowering pathway, further supporting this regulatory mechanism [24]. The consistency of this expression pattern between the two accessions further emphasizes the conserved role of *LuTPS6* genes in flowering time regulation. Interestingly, *LuTPS10.1* and *LuTPS10.2* also displayed substantial expression in both reproductive and vegetative tissues, with particularly high levels in the leaf and flower, respectively (Fig. 3a, b). In *Arabidopsis*, *TPS1* activity is essential for the induction of florigen *FT* in leaves, even during inductive photoperiods. *TPS1* facilitates the activation of *FT* by *CO* in response to increasing day length and high carbohydrate availability, as indicated by elevated T6P levels. This coordination signals that sufficient carbohydrate resources are available to support the energy-intensive processes of flowering and seed development [24].

Similarly, the expression of *LuTPS7* genes was notable across both reproductive and vegetative tissues, though *LuTPS7.1* and *LuTPS7.2* showed a bias toward reproductive tissues in both accessions (Fig. 3a, b). Notably, *LuTPS7.4* displayed high expression in leaf tissue, particularly in the IC0523807 accession, suggesting its significant role in flowering time regulation. Interestingly, the *LuTPS7.4* was identified as a candidate gene for flowering time regulation using genome-wide association study (GWAS) in linseed [39]. The high expression of *LuTPS7.4* in both reproductive and leaf tissues, coupled with its identification as a candidate gene for the flowering time trait, strongly suggests its potential role in regulating flowering time in linseed.

Towards identifying key flowering genes in linseed (flaxseed), House et al. [9] studied transcriptome of shoot apical meristem (SAM) from ‘Royal’ flax cultivar at 10, 15, 19, and 29 days after planting (dap). The dataset from this study was used to check expression of TPS genes in early developmental stages of floral transition. Interestingly, a similar pattern of gene expression was observed in the SAM, consistent with the findings of the present study, where the highest expression levels were detected for LuTPS6.1 and LuTPS6.2, followed by LuTPS10.1, LuTPS10.2, LuTPS7.4, LuTPS7.1, LuTPS7.2, LuTPS7.3, LuTPS1.1, and LuTPS1.2 (Figure S4). Moreover, both the studies show, little or no expression of LuTPS1.3, LuTPS1.4, LuTPS7.5, LuTPS7.6, and LuTPS11.1 (Fig. 3, Fig S4). The elevated expression of the mentioned TPS genes during the early stages of floral transition [9] and the later stages of floral development observed in the present study further indicate their potential involvement in the regulation of flowering in linseed.

### Allele mining of *LuTPS* genes

By sequencing and comparing four accessions comprising two early flowering-maturing (IC0523807, IC0525939) and two late flowering-maturing (EC0115148, EC0718827) accessions, multiple trait-specific SNPs were identified. In the *LuTPS6.1* gene, three SNPs were identified, all of which exhibited a clear trait-specific pattern, distinguishing between early and late flowering-maturing accessions (Table 2). In *LuTPS10.2,* the nonsynonymous SNP which resulted in an amino acid substitution Gly782Val (Glycine in late flowering-maturing accessions to Valine in early flowering-maturing accessions) at position 782 could particularly be noteworthy as glycine is a small, flexible amino acid, while valine is larger and more hydrophobic. Homology-based modeling of the *LuTPS10.2* protein revealed that this amino acid substitution leads to increased intramolecular interactions (Fig. 5), stabilizing the protein and potentially improving its functionality. Further, the analysis of potential energy showed that protein from early flowering-maturing accessions had lower potential energy compared to those from late flowering-maturing accessions (Table 4), indicating that the substitution likely contributes to protein stabilization. A single amino acid change in a protein has previously been shown to have a substantial effect not only on protein stability but also on its downstream function.

### Analysis of CSBs in linseed and other plant species

Classically, synteny refers to a set of loci in different species located on the same chromosome, though not necessarily in the same order. However, in the context of modern comparative genomics, synteny more commonly refers to conserved collinearity and genomic context, meaning the conservation of genes in the same order across two sets of chromosomes, either interspecific or intraspecific. Syntenic studies provide crucial insights into chromosomal rearrangements, the expansion and contraction of gene families, and gene orthology. Additionally, synteny can offer valuable information on gene function and regulation, often considered a measure of the conservation or alteration of gene function [70].

In the present study, genome scale synteny analysis of linseed and nine other plant species, including representatives from cereals, oilseeds, pulses, and the model plant *Arabidopsis*, provided critical insights into CSBs and their associated genes along with the GCNs. The highest number of CSBs at genome scale, as well as the *LuTPS* gene-containing CSBs, was found between linseed and dicot species such as soybean, sunflower, cowpea, and sesame. This suggests a greater conservation of linseed syntelogs with dicots compared to monocots (cereals), aligning with the evolutionary timelines. Fewer CSB or limited syntelogs of linseed with cereals, especially wheat and barley, could possibly be due to different rates of genome evolution through events like whole-genome duplication or polyploidization, transposable element activity, and extensive chromosomal rearrangements. A similar observation was reported in the synteny network analysis of MADS-box transcription factors, particularly in wheat and barley, where no syntenic regions of their MADS-box genes were identified with other plant genomes. In terms of linseed intraspecific CSBs, present study identified a total of 811 CSBs, indicating the occurrence of whole genome duplication events. The whole-genome assembly of flax at the pseudomolecule level, along with subsequent analyses also confirmed the occurrence of whole genome duplication events in flax [52]. It has been estimated that two genome duplication events occurred, one more recently at 3.7–6.8 million years ago and a more ancient one at 23.8–44.1 million years ago [52]. The abovementioned study identified a total of 911 CSBs, slightly more than the present study, likely due to differences in methods and criteria used. Nonetheless, both studies arrive at the same conclusion that there has been substantial whole genome duplication and chromosomal rearrangements in the linseed genome.

Analysis of the *LuTPS*-specific GCNs provided further insights into the relationships among linseed *TPS* genes and their duplication events. For example, *LuTPS1.1*, *LuTPS1.2*, *LuTPS1.3*, and *LuTPS1.4*, which are orthologous to *Arabidopsis TPS1*, clustered together in Cluster I (Fig. 7a). However, syntenic relations were only found between *LuTPS1.1* & *LuTPS1.2*, and *LuTPS1.3* & *LuTPS1.4*. There were no syntenic connections between *LuTPS1.1* & *LuTPS1.2* and *LuTPS1.3* & *LuTPS1.4*, though these pairs did exhibit syntenic relationships with *TPS* genes from other plants, suggesting that these pairs diverged earlier, followed by later duplication events leading to their respective paralogues. Similarly, *LuTPS6.1* and *LuTPS6.2* (Cluster II, Fig. 7b) showed no direct syntenic interaction with each other but did show syntenic relationships with *TPS* genes from other plants, indicating an ancient duplication event. Further, the Cluster II was found specific only to the dicots. Similar monocot- and eudicot-specific communities were also observed in the synteny network analysis of auxin response factor genes across diverse plant species. Other linseed *TPS* paralogues, such as *LuTPS7.5* and *LuTPS7.6* (Fig. 7a), demonstrated direct syntenic relations but lacked direct connections with *LuTPS7.1*—*LuTPS7.4*, suggesting earlier duplication and subsequent divergence. *LuTPS7.1*, *LuTPS7.2*, *LuTPS7.3*, and *LuTPS7.4*, on the other hand, exhibited syntenic connections among themselves, indicating more recent duplication events. These observations of genome duplication within *LuTPS*-specific GCNs align with findings of at least two WGD events in linseed [52]. Interestingly, the CSBs containing *LuTPS* genes also harbored important genes related to reproductive tissue development, photoperiodism, flowering, histone remodeling and modification, and protein ubiquitination. Genes participating in shared biological processes often cluster together genomically and are regulated by common promoters or signaling pathways. The close physical proximity of genes involved in such biological processes to *LuTPS* genes, particularly within the CSBs, suggests a potential involvement of *LuTPS* genes in these processes. However, further studies exploring the potential roles and associations of these genes with *LuTPS* genes would be required for deepening our understanding of flowering time regulation in linseed.

Potential protein–protein interaction of LuTPS, revealed a total of 27 unique interacting partners. The paralogues LuTPS showed similar interacting partners, possible due to their high sequence similarity. Most of the interacting partners were as per the expected function of TPS in sugar metabolism. However, three TPS, LuTPS6.1, LuTPS6.2, and LuTPS11.2 showed other TPS, LuTPS1.3 and LuTPS1.4 as interacting partners. Indeed, the protein–protein interaction among the TPS proteins have been demonstrated in rice. Furthermore, it is noteworthy that three of the PPI partners of TPS are components of the GCN, suggesting potential molecular interactions. While syntenic relationships do not necessarily correspond to PPIs, certain gene pairs are presumed to be conserved due to either transcriptional or/and functional relationship [75].

### Enriched CRE on linseed TPS promoter sequences

*CRE* are transcription factor (TF) binding sites located in the promoter regions of genes. By regulating when, where, and to what extent a gene is expressed, *CRE*s ensure that plant genes are activated or silenced in a highly specific and coordinated manner [76]. To understand how the expression of specific genes is regulated, it is crucial to identify *CREs* within promoter regions and assess their biological significance.

The trehalose-6-phosphate, besides being central to trehalose synthesis, plays a key role in sugar signaling, growth regulation, stress tolerance, and hormone interactions, establishing it as a critical regulator of plant physiology and development [19]. Therefore, it is crucial to understand the landscape of *TPS* promoters in order to identify significant *CRE* motifs associated with *TPS* genes. However, every gene promoter contains numerous *CRE* motifs, therefore, statistical application is critical to determine which among the motifs are significantly enriched [76, 77]. Identification of enriched *CREs* in *LuTPS* gene promoters, along with the annotation of associated transcription factors, provided significant insights into the biological processes in which *TPS* genes may be involved. Several enriched *CREs* in *TPS* promoters were associated with the transcription factors involved in floral and reproductive tissue development, flowering time regulation, and the transition from vegetative to reproductive growth. For example, *TF_motif_seq_0250*, one of the enriched *CREs*, was found to be the binding site of LAT52, which plays a key role in pollen hydration and pollen tube growth [79]. This motif was also observed in the promoter region of the tomato endo-beta-mannanase gene (LeMAN5), associated with anther and pollen development [80]. It is important to note that *TF_motif_seq_0250* was present in all the linseed TPS genes at least once (Table S3). *TF_motif_seq_0146* is a recognition sequence of *Arabidopsis* SUF4 (SUPPRESSOR of FRI 4), a zinc-finger-containing transcription factor having role in delayed flowering in *Arabidopsis*. SUF4 was shown to be responsible for higher expression of FLOWERING LOCUS C (FLC), a floral repressor in *Arabidopsis*. SUF4 physically bound to the FLC promoter as shown in a chromatin immunoprecipitation assay [81]. Other important motifs were *TF_motif_seq_0321,* and *TF_motif_seq_0481*, which are associated with regulation of light responsive genes such as RBCS, PHYA, RCA or circadian-regulated promoters, some of which are involved in flowering time regulation via photoperiod pathway [82–84]. Besides this, it is intriguing to note that a significant number of enriched *CREs* (15 of 32 enriched *CREs*) were associated with Dof-type domain-containing proteins. DOF proteins are known to repress CONSTANS expression and thus delay the flowering in *Arabidopsis* [85]. Mutations in DOF genes also contribute to photoperiod-insensitivity and early flowering in *Arabidopsis*, while overexpression of DOF genes from other plants showed delayed flowering [87]. It is noteworthy that the T6P pathway act under the photoperiod pathway as it integrates environmental signals (such as photoperiod) and physiological signals (such as carbohydrate status), and thereby regulate flowering by inducing FT in *Arabidopsis* [24]. Accordingly, the results indicate that the DOF proteins may possibly be involved in regulation of TPS gene expression under photoperiod pathway. Additionally, presence of six enriched *CREs* in all the linseed *TPS* genes (Table 5, Table S3), underscore their potential role in regulating TPS gene family expression through a common transcriptional regulatory mechanism.

### Co-location of LuTPS genes in close physical proximity of QTNs associated with flowering

In linseed, a total of 82 QTLs/QTNs/SNPs associated with the flowering trait have been reported so far by multiple studies [ 37–39, 88]. Interestingly, 9 out of the total 18 *LuTPS* genes were found within 1.0 Mb range of 12 QTLs/QTNs/SNPs. Among these, *LuTPS1.1*, was located 0.69, and 1.00 Mb away from the QTNs *Lu11_3283122*, and *Lu11_1592089*, respectively [39]. Further, *LuTPS1.3* was located 0.39 Mb distant from the QTN *Lu12_2899524* [37]. Another gene *LuTPS7.2*, was located 0.46, and 1.00 Mb away from the SNPs/QTNs *Lu12_2899524*, and *Lu12_4359290*, respectively [37, 39]. Gene *LuTPS7.3* could also be co-located 0.46 and 0.51 Mb distant from the SNPs/QTNs *Lu03_8373065*, and *Lu03_7400113*, respectively [37, 39]. Further, *LuTPS7.4,* a candidate gene identified earlier for flowering time trait was shown to harbor the associated QTN (*Lu15_2036006)* within the gene itself (39). Moreover, an additional QTN, *Lu15_1756429* was found only 0.28 Mb distant from *LuTPS7.4* gene. These findings underscore the strong candidature of *LuTPS7.4* for the flowering time trait in linseed. Gene *LuTPS7.6* was located just 0.83 Mb away from the SNP *Lu4_16669798* [37]. Further, gene *LuTPS8.1* was found located just 0.36 Mb distant from the SNP *Lu4_9221348* [37]. Gene *LuTPS8.2* could also be co-located 0.93 Mb away from the QTN *Lu01_6408072* [39]. Another gene, *LuTPS10.1* was found located 0.68 Mb away from the SNP *Lu13_17577340* [37]. These close physical proximities of key TPS genes to that of QTL/QTNs associated with flowering time trait suggest potential contribution of TPS genes towards flowering time regulation.

## Conclusion

This study offers comprehensive insights into the trehalose-6-phosphate synthase (TPS) gene family in linseed through genome scale comparative analysis. Enriched *CRE* in TPS promoters and associated syntelogs, along with allelic variation between early- and late-flowering genotypes, highlight the potential role of TPS genes in regulating flowering time. These findings represent an important step forward in establishing the potential role of *trehalose-6-phosphate synthase* genes in the complex regulation of flowering time in linseed.

## Supplementary Information

Supplementary Material 1.

Supplementary Material 2.

Supplementary Material 3.

Supplementary Material 4.

Supplementary Material 5.

Supplementary Material 6.

Supplementary Material 7.

Supplementary Material 8.

Supplementary Material 9.

## Data Availability

The datasets used in this study are available in the GenBank, NCBI repository, with Bio Project accession number, PRJNA773597, PRJNA1207411.
