# Supplementary figures and images for: Characterization of trehalose-6-phosphate synthase gene family in linseed (Linum usitatissimum L.) and its potential implications in flowering time regulation
Source: BMC Plant Biol. 2025 Nov 17;25:1581. doi: 10.1186/s12870-025-07559-7 (PMC12625084; doi:10.1186/s12870-025-07559-7)

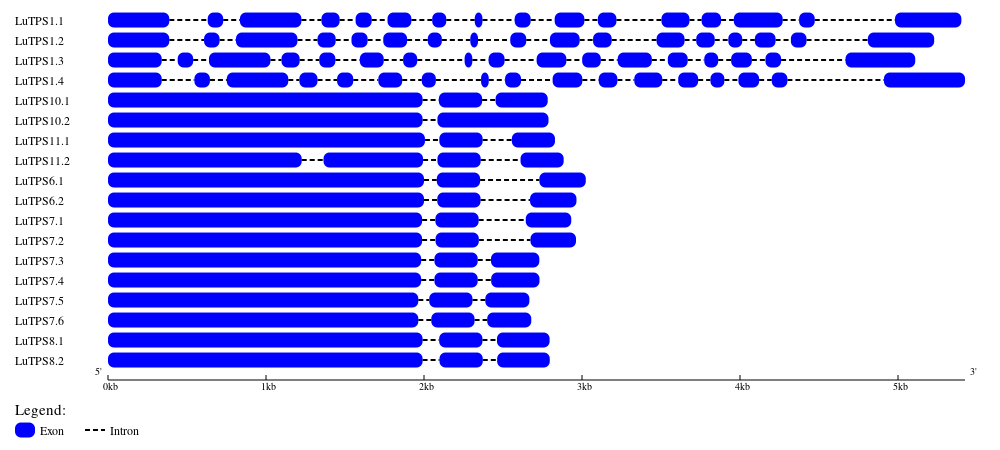

Supplement: Supplementary file 1 — Supplementary Material 1. [file 12870_2025_7559_MOESM1_ESM.png]

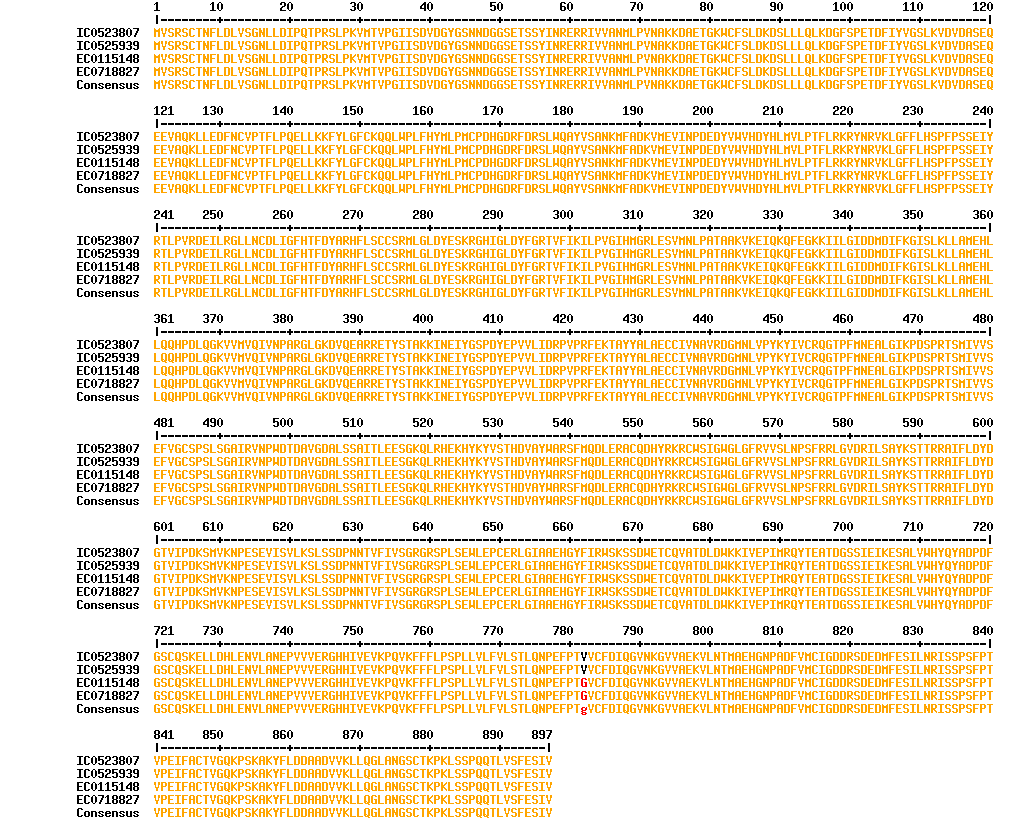

Supplement: Supplementary file 2 — Supplementary Material 2. [file 12870_2025_7559_MOESM2_ESM.gif]

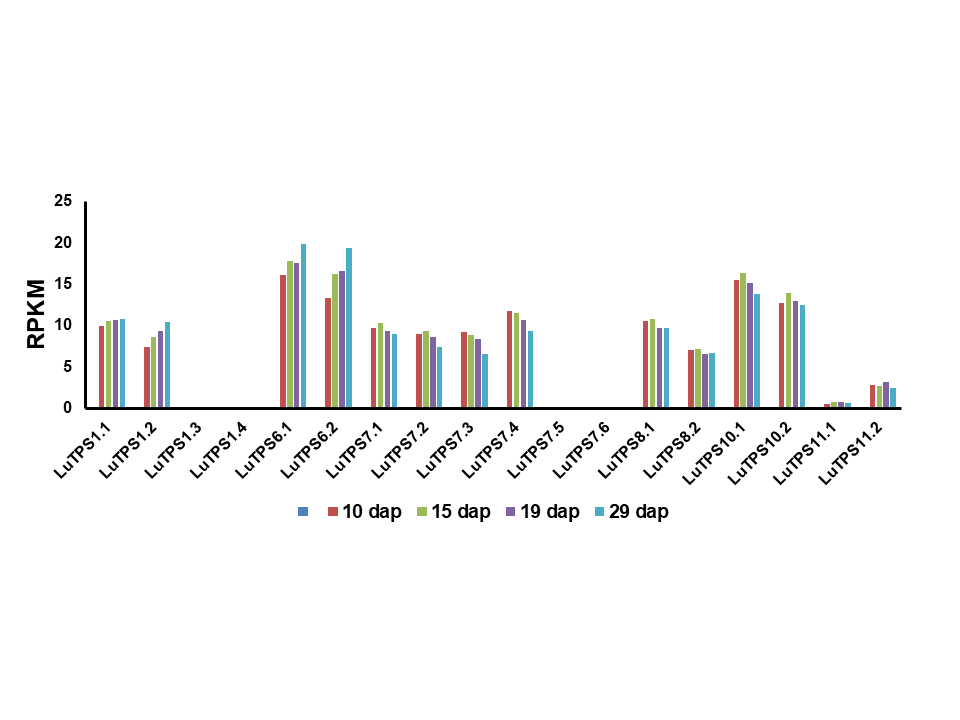

Supplement: Supplementary file 4 — Supplementary Material 4. [file 12870_2025_7559_MOESM4_ESM.png]
