# Supplementary material for: Characterization of trehalose-6-phosphate synthase gene family in linseed (Linum usitatissimum L.) and its potential implications in flowering time regulation
Source: BMC Plant Biol. 2025 Nov 17;25:1581. doi: 10.1186/s12870-025-07559-7 (PMC12625084; doi:10.1186/s12870-025-07559-7)
Supplement: Supplementary file 3 — Supplementary Material 3. [file 12870_2025_7559_MOESM3_ESM.docx]

**Figure S3.** Genome-wide synteny analysis of linseed with *Arabidopsis thaliana, Sesamum indicum, Hordeum vulgare, Oryza sativa, Triticum aestivum and Vigna radiata.* The genome wide conserved syntenic blocks (CSB) between the two species are depicted in grey shade, and the CSBs harbouring linseed TPS are shown with red lines.

***
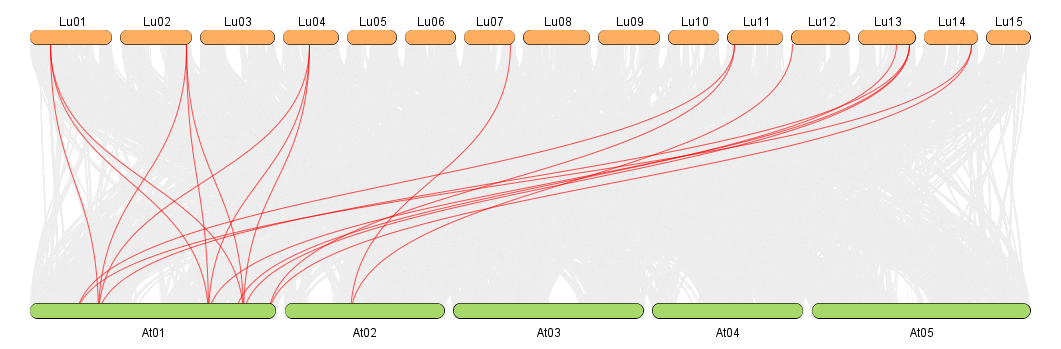
L. usitatissimum* and *Arabidopsis thaliana***

***L. usitatissimum and Sesamum indicum***

***
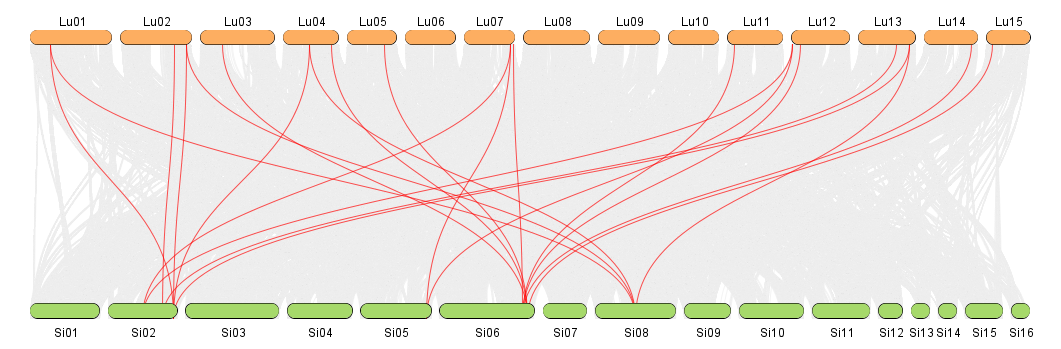
***

***
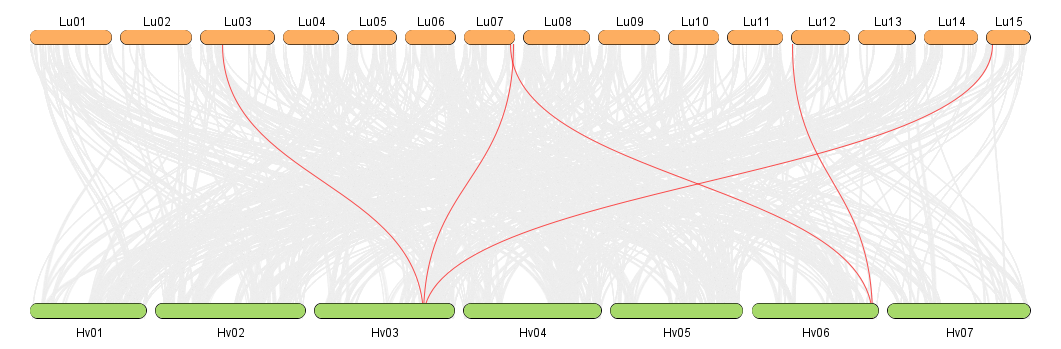
L. usitatissimum* and *Hordeum vulgare***

***
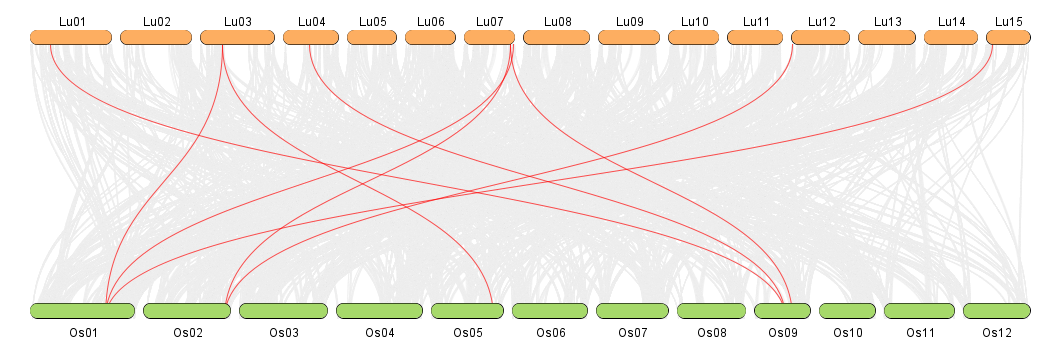
L. usitatissimum and Oryza sativa***

***L. usitatissimum and Triticum aestivum***

***
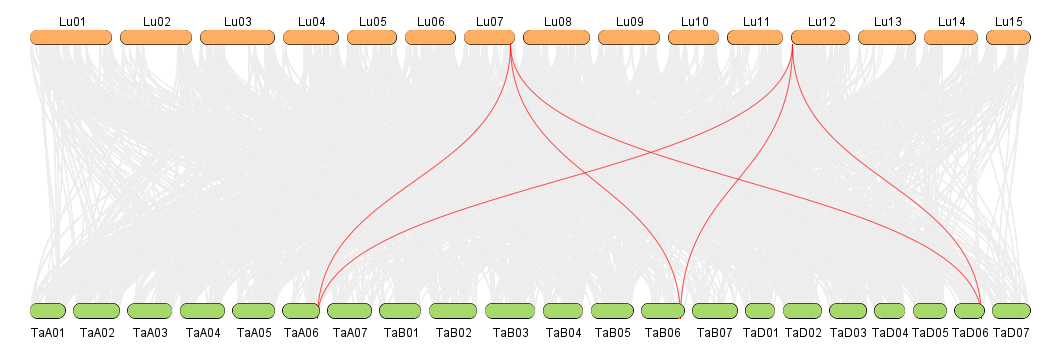
***

***L. usitatissimum and Vigna radiata***

***
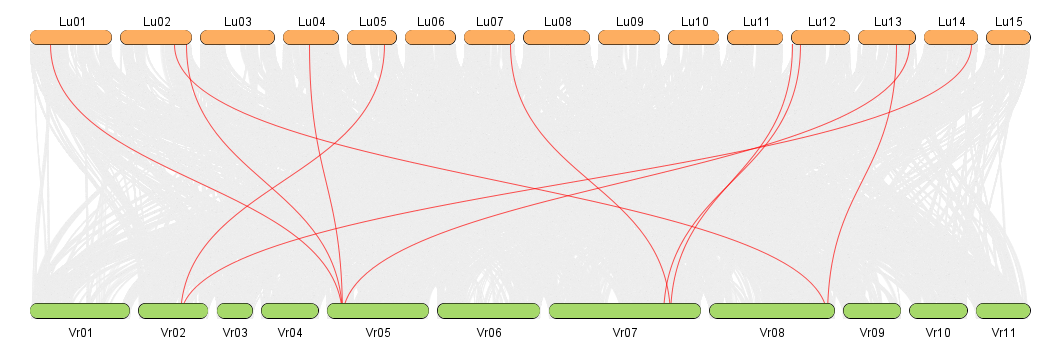
***
