# Supplementary material for: Characterization of trehalose-6-phosphate synthase gene family in linseed (Linum usitatissimum L.) and its potential implications in flowering time regulation
Source: BMC Plant Biol. 2025 Nov 17;25:1581. doi: 10.1186/s12870-025-07559-7 (PMC12625084; doi:10.1186/s12870-025-07559-7)
Supplement: Supplementary file 5 — Supplementary Material 5. [file 12870_2025_7559_MOESM5_ESM.docx]

**Supplementary Table 1:** List of 4 linseed germplasm accessions (2 early flowering-maturing, 2 late flowering-maturing) selected for allele mining of the *LuTPS* gene family.

| **Trait** | **Accession** | **DF50** | | **DM** | | **Geographical location** |
| --- | --- | --- | --- | --- | --- | --- |
|  |  | **2017-18** | **2018-19** | **2017-18** | **2018-19** |  |
| **Early flowering-maturing** | IC0523807 | 64.64 | 64.33 | 124.50 | 130.67 | Orissa, India |
|  | IC0525939 | 64.64 | 65.33 | 118.50 | 127.67 | Uttar Pradesh, India |
| **Late flowering-maturing** | EC0115148 | 113.81 | 99.33 | 149.17 | 150.83 | United State of America |
|  | EC0718827 | 126.81 | 122.33 | 155.17 | 153.83 | Australia |

DF50: Days to 50% flowering; DM: Days to maturity.
