# Supplementary material for: Characterization of trehalose-6-phosphate synthase gene family in linseed (Linum usitatissimum L.) and its potential implications in flowering time regulation
Source: BMC Plant Biol. 2025 Nov 17;25:1581. doi: 10.1186/s12870-025-07559-7 (PMC12625084; doi:10.1186/s12870-025-07559-7)
Supplement: Supplementary file 7 — Supplementary Material 7. [file 12870_2025_7559_MOESM7_ESM.docx]

**Table S3:** *Cis*-regulatory elements identified at least once within the 2 kb promoter regions of all LuTPS genes.

| **Matrix ID** | **Species** | **Matrix logo** | **Corresponding TF** | **Description** |
| --- | --- | --- | --- | --- |
| *TF_motif_seq_0237* | *A. thaliana* | 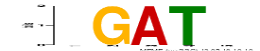 | *AT1G51600* | ZIM-LIKE 2; GATA transcription factor 28 (Protein TIFY 2A) |
| *TF_motif_seq_0239* | *A. thaliana* | 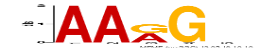 | *AT1G29160* | Dof-type zinc finger DNA-binding family protein |
| *TF_motif_seq_0240* | *A. thaliana* | 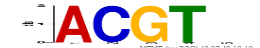 | *AT3G54620* | basic leucine zipper 25 (AtbZIP25); ACGT sequence |
| *TF_motif_seq_0241* | *A. thaliana* | 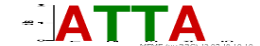 | *AT1G75240* | homeobox protein 33; Zinc-finger homeodomain protein 5 (AtZHD5) |
| *TF_motif_seq_0243* | *A. thaliana* | 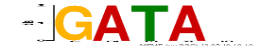 | *AT1G51600* | GATA box |
| *TF_motif_seq_0246* | *A. thaliana* | 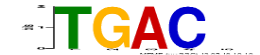 | *AT1G23380* | A core of TGAC-containing W-box |
| *TF_motif_seq_0252* | *A. thaliana* | 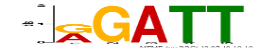 | *AT2G01760* | response regulator 1; Two-component response regulator ARR1 |
| *TF_motif_seq_0254* | *A. thaliana* | 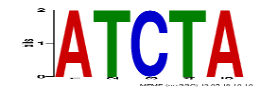 | *AT3G14230* | related to AP2 2; Ethylene-responsive transcription factor RAP2-2 |
| *TF_motif_seq_0255* | *A. thaliana* | 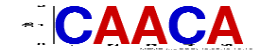 | *AT1G25560* | RAV1 ; RAV1 protein contain AP2-like and B3-like domains |
| *TF_motif_seq_0256* | *O. sativa* | 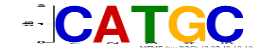 | *Os04g0676600* | ABI3/VP1 transcription factor family protein |
| *TF_motif_seq_0257* | *A. thaliana* | 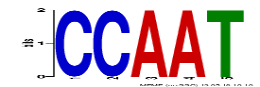 | *AT1G09030* | "CCAAT box" found in the promoter of heat shock protein genes |
| *TF_motif_seq_0258* | *A. thaliana* | 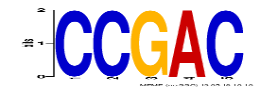 | *U01377* | Low temperature responsive element (LTRE), ABA responsiveness |
| *TF_motif_seq_0266* | *O. sativa* | 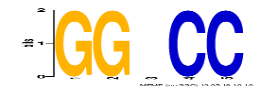 | *Os03g0785800* | Similar to Transcription factor PCF |
| *TF_motif_seq_0267* | *A. thaliana* | 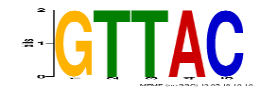 | *AT5G01380* | Homeodomain-like superfamily protein; Trihelix transcription factor GT-3a |
| *TF_motif_seq_0270* | *A. thaliana* | 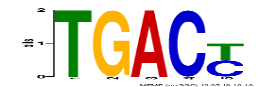 | *AT1G13960* | Probable WRKY transcription factor |
| *TF_motif_seq_0271* | *A. thaliana* | 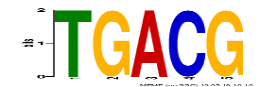 | *AT1G77920* | ASF-1 binding site in CaMV 35S promoter |
| *TF_motif_seq_0282* | *O. sativa* | 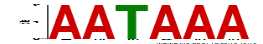 | *M24286* | Near upstream elements (NUE) in Arabidopsis |
| *TF_motif_seq_0300* | *A. thaliana* | 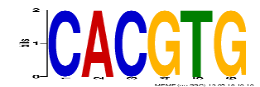 | *AT1G09530* | CACGTG motif; "G-box" |
| *TF_motif_seq_0302* | *A. thaliana* | 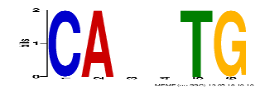 | *AT5G08130* | E-box of napA storage-protein gene of Brassica napus |
| *TF_motif_seq_0313* | *A. thaliana* | 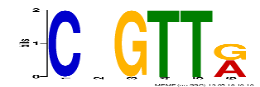 | *D14712* | Binding site for at least two plant MYBproteins |
| *TF_motif_seq_0316* | *O. sativa* | 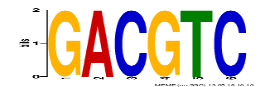 | *L345501* | C-box according to the nomenclature of ACGT elements |
| *TF_motif_seq_0327* | *O. sativa* | 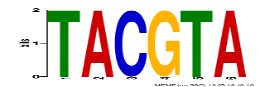 | *L34551* | A-box according to the nomenclature of ACGT elements |
| *TF_motif_seq_0337* | *P. sativum* | 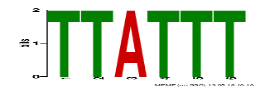 | *U22971* | TATA box |
| *TF_motif_seq_0339* | *A. thaliana* | 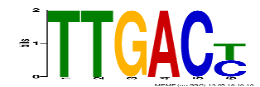 | *AT1G13960* | Probable WRKY transcription factor |
| *TF_motif_seq_0347* | *O. sativa* | 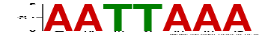 | *M24286* | PolyA signal; poly A signal found in rice alpha-amylase; -10 to-30 |
| *TF_motif_seq_0349* | *A. thaliana* | 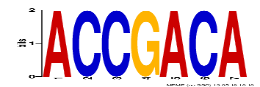 | *X67670* | Putative low temperature responsive element (LTRE) |
| *TF_motif_seq_0376* | *O. sativa* | 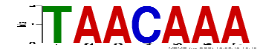 | *Os01g0812000* | Central element of gibberellin (GA) response complex (GARC) |
| *TF_motif_seq_0379* | *O. sativa* | 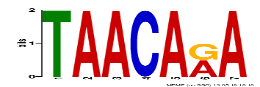 | *X16509* | amylase box |
| *TF_motif_seq_0410* | *A. thaliana* | 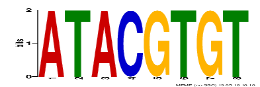 | *AT1G32640* | Z-DNA-forming sequence found in the Arabidopsis "Z-box" |
| *TF_motif_seq_0431* | *O. sativa* | 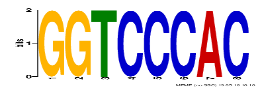 | *Os04g0194600* | Transcription factor PCF1, Transcription factor PCF2 |
| *TF_motif_seq_0508* | *A. thaliana* | 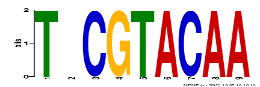 | *AT1G20980* | squamosa promoter binding protein |
| *TFmatrixID_0002* | *A. thaliana* | 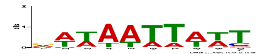 | *AT1G63480* | AT-HOOK MOTIF NUCLEAR LOCALIZED PROTEIN 12 |
| *TFmatrixID_0131* | *B. distachyon* | 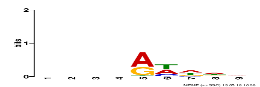 | *BRADI3G55950* |  |
| *TFmatrixID_0143* | *Z. mays* | 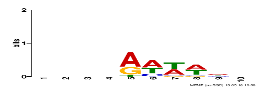 | *Zm00001d037159* |  |
| *TFmatrixID_0146* | *Z. mays* | 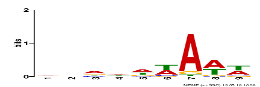 | *Zm00001d026348* |  |
| *TFmatrixID_0193* | *A. thaliana* | 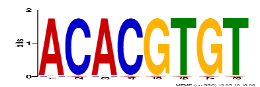 | *AT4G34000* | ABA-responsive element (ABRE). Stress-responsive ABA signalling. |
| *TFmatrixID_0223* | *C. sativus* | 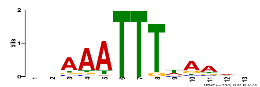 | *PK22848.1* | TCR family transcription factor |
| *TFmatrixID_0230* | *C. vulgaris* | 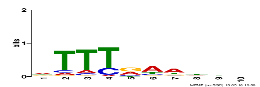 | *fgeneshCV_pg.C_scaffold_18000164* | TCR family transcription factor |
| *TFmatrixID_0235* | *A. thaliana* | 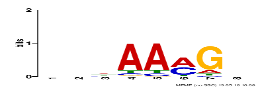 | *AT3G21270* | DOF zinc finger protein 2; Dof zinc finger protein DOF3.1 |
| *TFmatrixID_0243* | *A. thaliana* | 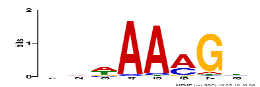 | *AT5G62940* | Dof zinc finger protein DOF5.6 |
| *TFmatrixID_0296* | *O. sativa* | 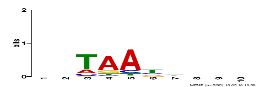 | *Os02g0706600* | Similar to zinc finger homeodomain protein 1 |
| *TFmatrixID_0580* | *O. sativa* | 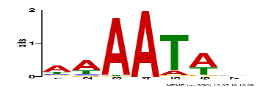 | *Os01g0835600* |  |
| *TFmatrixID_0992* | *A. thaliana* | 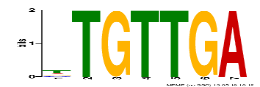 | *AT5G42820* | Necessary for the splicing of pre-mRNA (By similarity). |
| *TFmatrixID_0994* | *A. thaliana* | 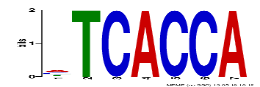 | *AT1G70910* | RING/U-box superfamily protein |
| *TFmatrixID_1369* | *A. thaliana* | 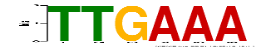 | *AT1G63040* | Probably acts as a transcriptional activator. GCC-box |
| *TFmatrixID_1528* | *A. thaliana* | 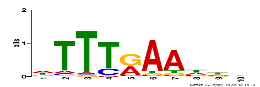 | *AT3G22760* | Tesmin/TSO1-like CXC domain-containing protein |
| *TFmatrixID_1539* | *A. thaliana* | 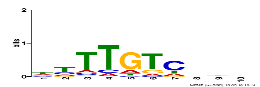 | *AT3G50700* | indeterminate (ID)-domain 2 |
| *TF_motif_seq_0003* | *S. tuberosum* | 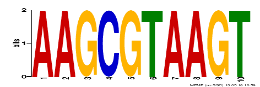 |  | Binding site of wound-inducible nuclear protein |
| *TF_motif_seq_0009* | *A. thaliana* | 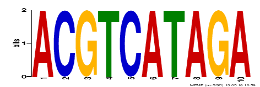 |  | A positive salicylic acid-inducible element |
| *TF_motif_seq_0148* | *P. vulgaris* | 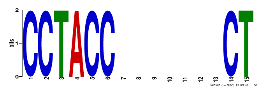 |  | H-box. |
| *TF_motif_seq_0238* | *Z. mays* | 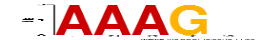 |  | Core site required for binding of Dof proteins in maize (Z.m.) |
| *TF_motif_seq_0242* | *P. sativum* | 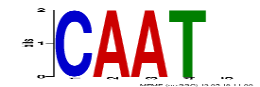 |  | CAAT promoter consensus sequence found in legA gene of pea. |
| *TF_motif_seq_0245* | *N. tabacum* | 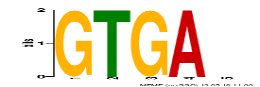 |  | GTGA motif found in the promoter of the tobacco |
| *TF_motif_seq_0247* | *F. trinervia* | 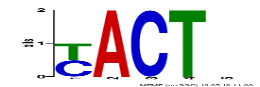 |  | Tetranucleotide (CACT) |
| *TF_motif_seq_0248* | *A. thaliana* | 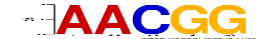 |  | Myb core in the 18 bp sequence |
| *TF_motif_seq_0249* | *A. thaliana* | 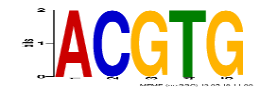 |  | ABRE-like sequence (from -199 to -195) |
| *TF_motif_seq_0250* | *L. esculentum* | 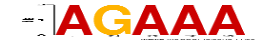 |  | regulatory elements for pollen specific activation of tomato lat52 gene. |
| *TF_motif_seq_0253* | *A. rhizogenes* | 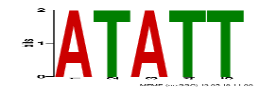 |  | Motif found both in promoters of rolD. |
| *TF_motif_seq_0259* | *O. sativa* | 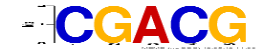 |  | CGACG element found in the rice Amy3D and Amy3E amylase genes. |
| *TF_motif_seq_0260* | *V. faba* | 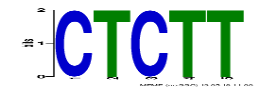 |  | MADS box |
| *TF_motif_seq_0261* | *A. thaliana* | 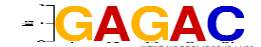 |  | Core of sulfur-responsive element (SURE) found in the SULTR1.1 |
| *TF_motif_seq_0262* | *-* | 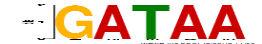 |  | I box. "I-box". Conserved sequence upstream of light-regulated genes. |
| *TF_motif_seq_0263* | *A. thaliana* | 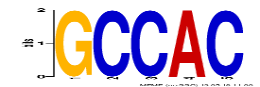 |  | one of "Sequences Over-Represented in Light-Induced Promoters |
| *TF_motif_seq_0264* | *S. tuberosum* | 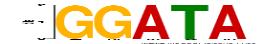 |  | Core motif of MybSt1 (a potato MYB homolog) binding site. |
| *TF_motif_seq_0265* | *A. thaliana* | 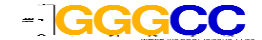 |  | one of "Sequences Over-Represented in Light-Induced Promoters |
| *TF_motif_seq_0268* | *A. thaliana* | 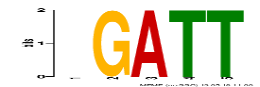 |  | ARR1-binding element found in Arabidopsis. |
| *TF_motif_seq_0269* | *S. tuberosum* | 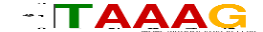 |  | TAAAG motif. Target site for trans-acting StDof1 protein |
| *TF_motif_seq_0272* | *H. vulgare* | 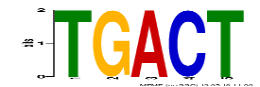 |  | W-box element in barley iso1 (encodingisoamylase1) promoter. |
| *TF_motif_seq_0273* | *N. tabacum* | 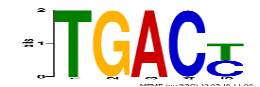 |  | W box found in the promoter region of a transcriptional repressor ERF3 |
| *TF_motif_seq_0274* | *O. sativa* | 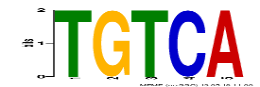 |  | Binding site of OsBIHD1. a rice BELL homeodomain transcription factor. |
| *TF_motif_seq_0275* | *A. thaliana* | 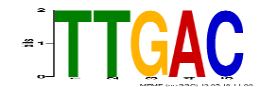 |  | W-box found in promoter of Arabidopsis thaliana NPR1gene. |
| *TF_motif_seq_0280* | *(Viridiplantae)* | 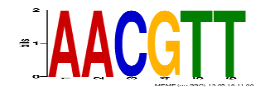 |  | T-box according to the nomenclature of ACGT elements |
| *TF_motif_seq_0283* | *Z. mays* | 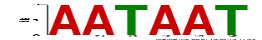 |  | Plant polyA signal. Consensus sequence for plantpolyadenylation signal. |
| *TF_motif_seq_0315* | *G. max* | 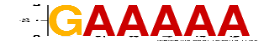 |  | GT-1 motif found in the promoter of Glycine max. SCaM-4. |
| *TF_motif_seq_0321* | *P. sativum* | 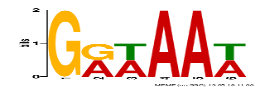 |  | Consensus GT-1 binding site in many light-regulated genes. |
| *TF_motif_seq_0322* | *H. vulgare* | 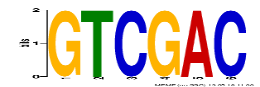 |  | Preferred sequence for AP2 transcriptional activator HvCBF2 of barley. |
| *TF_motif_seq_0341* | *A. thaliana* | 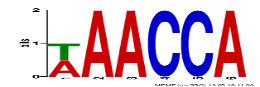 |  | MYB recognition site in the promoters of the dehydration-responsive rd22 |
| *TF_motif_seq_0343* | *Z. mays* | 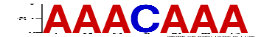 |  | One of 16 motifs found in silico in promoters of 13 anaerobic genes |
| *TF_motif_seq_0344* | *O. sativa* | 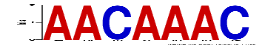 |  | Core of AACA motifs found in rice glutelin genes |
| *TF_motif_seq_0345* | *B. napus* | 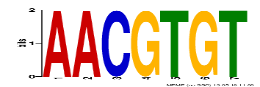 |  | Quantitative activator region in promoter of Brassica napus extensin gene. |
| *TF_motif_seq_0346* | *L. esculentum* | 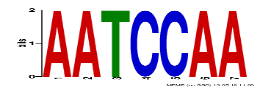 |  | rbcS general consensus sequence. AATCCAA or AATCCAAC. |
| *TF_motif_seq_0350* | *Z. mays* | 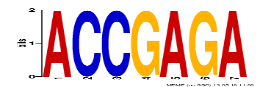 |  | DRE1 core found in maize (Z.M.) rab17 gene promoter. |
| *TF_motif_seq_0357* | *B. napus* | 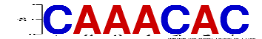 |  | Conserved in many storage-protein gene promoters. |
| *TF_motif_seq_0364* | *C. reinhardtii* | 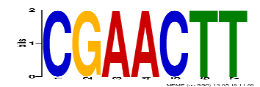 |  | Motif (IVD) found in the Chlamydomonas (C.R.) Nia1 gene promoter. |
| *TF_motif_seq_0370* | *C. reinhardtii* | 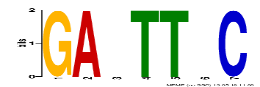 |  | Consensus motif of the two enhancer elements. EE-1 andEE-2. |
| *TF_motif_seq_0375* | *G. max* | 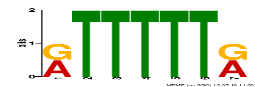 |  | 5'upstream region (-199) of Soybean Embryo Factor 4 (SEF4) gene |
| *TF_motif_seq_0377* | *A. thaliana* | 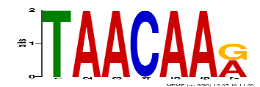 |  | GARE (GA-responsive element). |
| *TF_motif_seq_0380* | *O. sativa* | 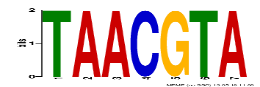 |  | Gibberellin-responsive element (GARE) |
| *TF_motif_seq_0383* | *P. sativum* | 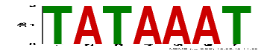 |  | TATA box. |
| *TF_motif_seq_0384* | *I. batatas* | 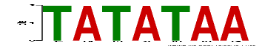 |  | TATA box |
| *TF_motif_seq_0388* | *I. batatas* | 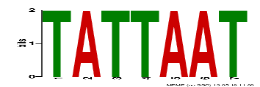 |  | TATA box. |
| *TF_motif_seq_0389* | *O. sativa* | 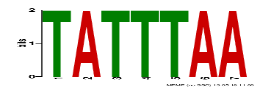 |  | Binding site for OsTBP2. found in the promoter of rice pal gene |
| *TF_motif_seq_0399* | *A. thaliana* | 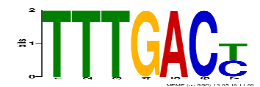 |  | W box. WRKY proteins bind specifically to motif (T)(T)TGAC(C/T). |
| *TF_motif_seq_0407* | *I. batatas* | 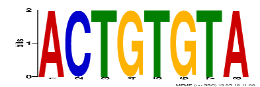 |  | One of SPBF binding site (SP8a). |
| *TF_motif_seq_0413* | *N. tabacum* | 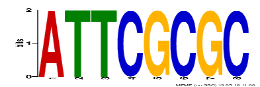 |  | pE2F (proximal E2F elemen) at -143bp of tobacco (N.t.) RNR1apromoter. |
| *TF_motif_seq_0414* | *L. esculentum* | 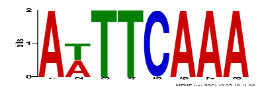 |  | ERE (ethylene responsive element) of tomato E4 & carnation GST1 genes. |
| *TF_motif_seq_0429* | *O. sativa* | 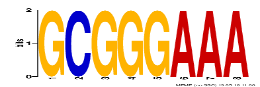 |  | re2f-1 found in the promoter of rice PCNA gene. |
| *TF_motif_seq_0432* | *G. max* | 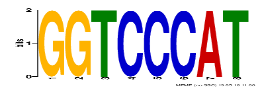 |  | Sequence found in NDE element in Soybean |
| *TF_motif_seq_0449* | *G. max* | 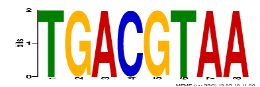 |  | TGA-box #1 in putative auxin-resonsive element (AUXRE) of soybean |
| *TF_motif_seq_0450* | *G. max* | 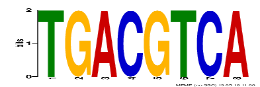 |  | Palindromic C-box in soybean |
| *TF_motif_seq_0453* | *H. vulgare* |  |  | Endosperm motif (EM) found in the promoter of barley c-hordein gene. |
| *TF_motif_seq_0455* | *N. tabacum* |  |  | E2Fa element found in the tobacco RNR (Ribonucleotidereductase) gene |
| *TF_motif_seq_0458* | *N. tabacum* |  |  | E2F consensus sequence of all different E2F-DP-binding motifs |
| *TF_motif_seq_0460* | *N. tabacum* |  |  | Initiator elements-tobacco psaDb promoter without TATA boxes. |
