# Supplementary material for: Characterization of trehalose-6-phosphate synthase gene family in linseed (Linum usitatissimum L.) and its potential implications in flowering time regulation
Source: BMC Plant Biol. 2025 Nov 17;25:1581. doi: 10.1186/s12870-025-07559-7 (PMC12625084; doi:10.1186/s12870-025-07559-7)
Supplement: Supplementary file 9 — Supplementary Material 9. [file 12870_2025_7559_MOESM9_ESM.pdf]

Gene ID	Description (Estimated PPV, Description)	Biological process (Estimated PPV, GO-id, Description)	Molecular function (Estimated PPV, GO-id, Description)	Cellular component (Estimated PPV, GO-id, Description)	Inverse ec2go, Kegg2go
Lus10019666	0.57 Trehalose 6-phosphate phosphatase	0.78 GO:0005992 trehalose biosynthetic process<br>0.40 GO:0009987 cellular process	0.82 GO:0004805 trehalose-phosphatase activity	0.32 GO:0016020 membrane	0.82 EC:3.1.3.12 GO:0004805
Lus10016657	0.83 Sucrose-phosphate synthase	0.83 GO:0005986 sucrose biosynthetic process<br>0.40 GO:0009987 cellular process	0.85 GO:0046524 sucrose-phosphate synthase activity<br>0.85 GO:0016157 sucrose synthase activity		0.85 EC:2.4.1.14 GO:0046524
Lus10016640	0.65 UDP-glucuronate 4-epimerase 6	0.49 GO:0033481 galacturonate biosynthetic process<br>0.44 GO:0050829 defense response to Gram-negative bacterium<br>0.43 GO:0050832 defense response to fungus	0.64 GO:0016853 isomerase activity<br>0.34 GO:0005515 protein binding	0.44 GO:0016020 membrane<br>0.40 GO:0005794 Golgi apparatus<br>0.35 GO:0005768 endosome<br>0.34 GO:0031984 organelle subcompartment	0.64 EC:5.-.-.- GO:0016853
Lus10008056	0.79 Sucrose-phosphate synthase	0.83 GO:0005986 sucrose biosynthetic process<br>0.40 GO:0009987 cellular process	0.85 GO:0046524 sucrose-phosphate synthase activity<br>0.81 GO:0016157 sucrose synthase activity<br>0.41 GO:0030246 carbohydrate binding		0.85 EC:2.4.1.14 GO:0046524
Lus10012454	0.71 Sucrose synthase	0.80 GO:0005985 sucrose metabolic process<br>0.40 GO:0009987 cellular process	0.85 GO:0016157 sucrose synthase activity		0.85 EC:2.4.1.13 GO:0016157
Lus10017984	0.0 Uncharacterized protein				
Lus10038739	0.52 Hexosyltransferase	0.78 GO:0045489 pectin biosynthetic process<br>0.67 GO:0071555 cell wall organization	0.85 GO:0047262 polygalacturonate 4-alpha-galacturonosyltransferase activity	0.70 GO:0000139 Golgi membrane	0.85 EC:2.4.1.43 GO:0047262
Lus10019010	0.57 Trehalose 6-phosphate phosphatase	0.78 GO:0005992 trehalose biosynthetic process<br>0.40 GO:0009987 cellular process	0.82 GO:0004805 trehalose-phosphatase activity<br>0.34 GO:0016757 glycosyltransferase activity		0.82 EC:3.1.3.12 GO:0004805
Lus10042456	0.92 Inactive glucose-1-phosphate adenylyltransferase small subunit, chloroplastic	0.76 GO:0005978 glycogen biosynthetic process<br>0.38 GO:0009231 riboflavin biosynthetic process<br>0.36 GO:0019252 starch biosynthetic process	0.80 GO:0008878 glucose-1-phosphate adenylyltransferase activity<br>0.54 GO:0005524 ATP binding<br>0.40 GO:0000906 6,7-dimethyl-8-ribityllumazine synthase activity<br>0.34 GO:0003677 DNA binding	0.40 GO:0009349 riboflavin synthase complex<br>0.34 GO:0009507 chloroplast	0.80 EC:2.7.7.27 GO:0008878<br>0.80 KEGG:R00948 GO:0008878

Lus10041979	0.51	Sucrose synthase	0.80 GO:0005985 sucrose metabolic process<br>0.40 GO:0009987 cellular process	0.85 GO:0016157 sucrose synthase activity	0.37 GO:0016020 membrane	0.85 EC:2.4.1.13 GO:0016157
Lus10041971	0.57	UTP--glucose-1-phosphate uridylyltransferase	0.77 GO:0006011 UDP-glucose metabolic process<br>0.57 GO:0006412 translation<br>0.41 GO:0005977 glycogen metabolic process	0.79 GO:0003983 UTP:glucose-1-phosphate uridylyltransferase activity<br>0.59 GO:0003735 structural constituent of ribosome	0.57 GO:0005840 ribosome<br>0.54 GO:1990904 ribonucleoprotein complex<br>0.37 GO:0098798 mitochondrial protein-containing complex<br>0.37 GO:0005759 mitochondrial matrix	0.79 EC:2.7.7.9 GO:0003983<br>0.79 KEGG:R00289 GO:0003983
Lus10016069	0.56	Glucose-1-phosphate adenylyltransferase	0.83 GO:0019252 starch biosynthetic process<br>0.76 GO:0005978 glycogen biosynthetic process	0.81 GO:0008878 glucose-1-phosphate adenylyltransferase activity<br>0.56 GO:0005524 ATP binding	0.66 GO:0009507 chloroplast	0.81 EC:2.7.7.27 GO:0008878<br>0.81 KEGG:R00948 GO:0008878
Lus10003045	0.55	Hexosyltransferase	0.78 GO:0045489 pectin biosynthetic process<br>0.68 GO:0071555 cell wall organization	0.85 GO:0047262 polygalacturonate 4-alpha-galacturonosyltransferase activity	0.71 GO:0000139 Golgi membrane	0.85 EC:2.4.1.43 GO:0047262
Lus10026213	0.50	ADP-glucose pyrophosphorylase small subunit 2	0.76 GO:0005978 glycogen biosynthetic process<br>0.37 GO:0009231 riboflavin biosynthetic process<br>0.36 GO:0019252 starch biosynthetic process	0.80 GO:0008878 glucose-1-phosphate adenylyltransferase activity<br>0.54 GO:0005524 ATP binding<br>0.38 GO:0000906 6,7-dimethyl-8-ribityllumazine synthase activity<br>0.33 GO:0003677 DNA binding	0.38 GO:0009349 riboflavin synthase complex<br>0.33 GO:0016020 membrane	0.80 EC:2.7.7.27 GO:0008878<br>0.80 KEGG:R00948 GO:0008878
Lus10000687	0.56	Trehalose 6-phosphate phosphatase	0.78 GO:0005992 trehalose biosynthetic process<br>0.40 GO:0009987 cellular process	0.82 GO:0004805 trehalose-phosphatase activity	0.32 GO:0016020 membrane	0.82 EC:3.1.3.12 GO:0004805
Lus10010088	0.55	Glucose-1-phosphate adenylyltransferase	0.83 GO:0019252 starch biosynthetic process<br>0.76 GO:0005978 glycogen biosynthetic process<br>0.36 GO:0060320 rejection of self pollen	0.81 GO:0008878 glucose-1-phosphate adenylyltransferase activity<br>0.56 GO:0005524 ATP binding	0.66 GO:0009507 chloroplast<br>0.36 GO:0009501 amyloplast<br>0.34 GO:0005576 extracellular region	0.81 EC:2.7.7.27 GO:0008878<br>0.81 KEGG:R00948 GO:0008878
Lus10023553	0.56	Glucose-1-phosphate adenylyltransferase	0.82 GO:0019252 starch biosynthetic process<br>0.76 GO:0005978 glycogen biosynthetic process	0.80 GO:0008878 glucose-1-phosphate adenylyltransferase activity<br>0.55 GO:0005524 ATP binding	0.65 GO:0009507 chloroplast	0.80 EC:2.7.7.27 GO:0008878<br>0.80 KEGG:R00948 GO:0008878

Lus10024607	0.58	Trehalose 6-phosphate phosphatase	0.78 GO:0005992 trehalose biosynthetic process<br>0.40 GO:0009987 cellular process	0.82 GO:0004805 trehalose-phosphatase activity		0.82 EC:3.1.3.12 GO:0004805
Lus10038119	0.81	Sucrose-phosphate synthase	0.83 GO:0005986 sucrose biosynthetic process<br>0.45 GO:0071836 nectar secretion<br>0.40 GO:0009987 cellular process	0.85 GO:0046524 sucrose-phosphate synthase activity<br>0.84 GO:0016157 sucrose synthase activity<br>0.39 GO:0030246 carbohydrate binding		0.85 EC:2.4.1.14 GO:0046524
Lus10022570	0.39	sucrose-phosphate synthase		0.85 GO:0046524 sucrose-phosphate synthase activity		0.85 EC:2.4.1.14 GO:0046524
Lus10022552	0.71	UDP-glucuronate 4-epimerase 6	0.53 GO:0033481 galacturonate biosynthetic process<br>0.47 GO:0050829 defense response to Gram-negative bacterium<br>0.46 GO:0050832 defense response to fungus	0.64 GO:0016853 isomerase activity<br>0.34 GO:0005515 protein binding	0.44 GO:0016020 membrane<br>0.42 GO:0005794 Golgi apparatus<br>0.35 GO:0005768 endosome<br>0.34 GO:0031984 organelle subcompartment	0.64 EC:5.-.-.- GO:0016853
Lus10007209	0.55	Glucose-1-phosphate adenylyltransferase	0.83 GO:0019252 starch biosynthetic process<br>0.76 GO:0005978 glycogen biosynthetic process<br>0.36 GO:0060320 rejection of self pollen	0.81 GO:0008878 glucose-1-phosphate adenylyltransferase activity<br>0.56 GO:0005524 ATP binding	0.66 GO:0009507 chloroplast<br>0.36 GO:0009501 amyloplast<br>0.34 GO:0005576 extracellular region	0.81 EC:2.7.7.27 GO:0008878<br>0.81 KEGG:R00948 GO:0008878
Lus10025187	0.56	Glucose-1-phosphate adenylyltransferase	0.83 GO:0019252 starch biosynthetic process<br>0.76 GO:0005978 glycogen biosynthetic process	0.81 GO:0008878 glucose-1-phosphate adenylyltransferase activity<br>0.56 GO:0005524 ATP binding	0.66 GO:0009507 chloroplast	0.81 EC:2.7.7.27 GO:0008878<br>0.81 KEGG:R00948 GO:0008878
Lus10032244	0.58	Trehalose 6-phosphate phosphatase	0.78 GO:0005992 trehalose biosynthetic process<br>0.40 GO:0009987 cellular process	0.82 GO:0004805 trehalose-phosphatase activity		0.82 EC:3.1.3.12 GO:0004805
Lus10033245	0.74	Starch synthase, chloroplastic/amyloplastic	0.83 GO:0019252 starch biosynthetic process	0.81 GO:0004373 alpha-1,4-glucan glucosyltransferase (UDP-glucose donor) activity<br>0.36 GO:0009011 alpha-1,4-glucan glucosyltransferase (ADP-glucose donor) activity	0.75 GO:0009501 amyloplast<br>0.60 GO:0009507 chloroplast<br>0.43 GO:0043036 starch grain	0.81 EC:2.4.1.11 GO:0004373
Lus10008279	0.75	Starch synthase, chloroplastic/amyloplastic	0.83 GO:0019252 starch biosynthetic	0.81 GO:0004373 alpha-1,4-glucan glucosyltransferase	0.75 GO:0009501 amyloplast<br>0.60 GO:0009507 chloroplast	0.81 EC:2.4.1.11 GO:0004373

		process	(UDP-glucose donor) activity<br>0.36 GO:0009011 alpha-1,4-glucan glucosyltransferase (ADP-glucose donor) activity	0.43 GO:0043036 starch grain	
Lus10039339	0.57 Trehalose 6-phosphate phosphatase	0.78 GO:0005992 trehalose biosynthetic process<br>0.40 GO:0009987 cellular process	0.82 GO:0004805 trehalose-phosphatase activity<br>0.34 GO:0016757 glycosyltransferase activity		0.82 EC:3.1.3.12 GO:0004805
